# Supplementary material for: High-resolution, large field-of-view label-free imaging via aberration-corrected, closed-form complex field reconstruction
Source: Nat Commun. 2024 Jun 3;15:4713. doi: 10.1038/s41467-024-49126-y (PMC11148160; doi:10.1038/s41467-024-49126-y)
Supplement: Supplementary file 1 — Supplementary Information [file 41467_2024_49126_MOESM1_ESM.pdf]

High-resolution, large field-of-view label-free imaging via aberration-corrected, closed-form complex field reconstruction: Supplementary Notes

Ruizhi Cao[1,*,†], Cheng Shen[1,†], and Changhuei Yang[1]

[1]Department of Electrical Engineering, California Institute of Technology, Pasadena, CA, USA
[*]rcao@caltech.edu
[†]These authors contribute equally to this work

Contents

1 System calibration **2**

2 Result of FPM and APIC using reduced dataset **3**

3 Resolution quantification **5**

4 Reconstruction of a hematoxylin and eosin stained sample **6**

5 Result of FPM and APIC when imaging a phase target **7**

6 Reconstruction time **8**

7 Aberration correction **9**

8 Comparison under different signal-to-noise ratios **11**

9 Number of NA-matching measurements required in APIC **14**

10 Inaccurate illumination angle estimates **15**

11 Result of spatial-domain Kramers-Kronig method and APIC **16**

12 Derivation of APIC **17**
12.1 The forward model 17
12.2 Reconstruction under the NA-matching angle illumination 18
12.3 Aberration extraction 22
12.4 Reconstruction using darkfield measurements 25

1 System calibration

To do reconstruction using the Angular Ptychograhic Imaging with Closed-form method (APIC), we need to know the transverse illumination vector $\mathbf{k}_i$ for each measurement, as it tells us which area of the whole sample's spectrum is measured (Eq. 6). This indicates that we need to determine the angle of each tilted illumination. To do that, we used the previously developed circle-finding algorithm to find the exact illumination angle for the NA-matching measurements [1]. The brightfield measurements whose illumination angles were below the acceptance angle of our imaging system were collected as well. These brightfield measurements were used for geometrically calibrating the angles associated with our darkfield measurements. We note that these brightfield measurements are only for calibration purpose and not for reconstruction in APIC.

The illumination unit consisted of a LED ring and a LED array. The LED ring was attached on top of the LED array and was used for the NA-matching measurement. This unit was mounted on a motorized transnational stage for height adjustment. We adjusted its tilt and height to exactly match the illumination angle of the ring LED and the acceptance angle of our imaging system. To find the exact height, we first moved the LED unit close to our sample such that the ring LED produced the darkfield measurement. Then, we gradually increased the separation between the LED and the sample until we saw the image under the ring LED illumination transited from darkfield to brightfield. The transition point is our desired height. Once the height and tilt of the system were fixed, we acquired all calibration data and calibrated the illumination angles for all LEDs. We also used a high NA objective to measure the relative intensity of each LED with a blank slide. The high NA objective was selected such that the incident light from any LED can directly enter the system. It needs to be emphasized that the intensity calibration is done only once with a high NA, small field-of-view objective, we acquired all our actual experiment data with a low NA, large field-of-view objective. In our experiment, we normalized the measurements using the measured relative intensities and then conducted the reconstruction in APIC.

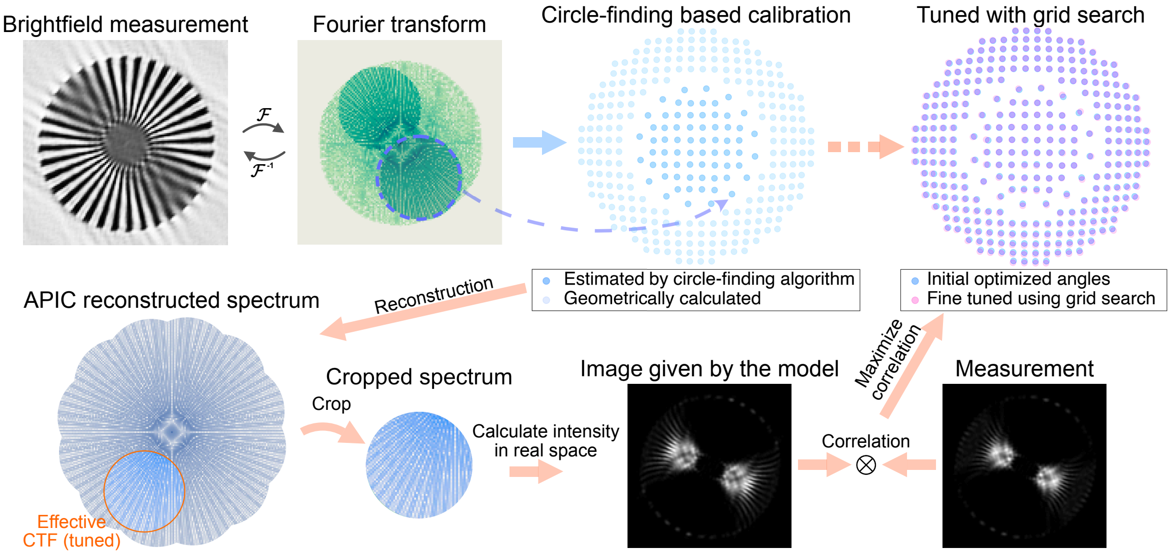

Figure S1: Calibrating the illumination k-vector. By locating the center of the circle in the Fourier transform of the measurement, we extract the corresponding $\mathbf{k}_i$ in the spatial frequency domain. Using the separation of the LEDs on the array and the estimated brightfield LED illumination angles, we can calculate the darkfield LED illumination angles using such geometry. With those, APIC could reconstruct sample's complex field, which can be used to further optimize the illumination angles by maximizing the correlation between the real measurement and the image obtained with the forward model. CTF: coherent transfer function.

We note that we can reconstruct the calibration data with the geometrically calculated darkfield LED illumination angle and then use the reconstructed complex field to further optimize the illumination angle by searching over a pre-defined finer grid. Once this is done, we fix the calibrated angles and use them for all other measurements. The entire process is illustrated in Fig. S1.

2 Result of FPM and APIC using reduced dataset

In our main manuscript, we acquired 316 images for one sample. As we explained in our main manuscript, this large redundancy was chosen to show the best performance of Fourier Ptychographic Microscopy (FPM). Here, we reduced the dataset so that there are 9 bright field measurements, 8 NA-matching measurements and 28 darkfield measurements in this reduced dataset. In our reconstruction, FPM used all these 45 images while APIC used 36 images (8 NA-matching measurements and 28 darkfield measurements). The arrangement of the LEDs is shown in Fig. S2.

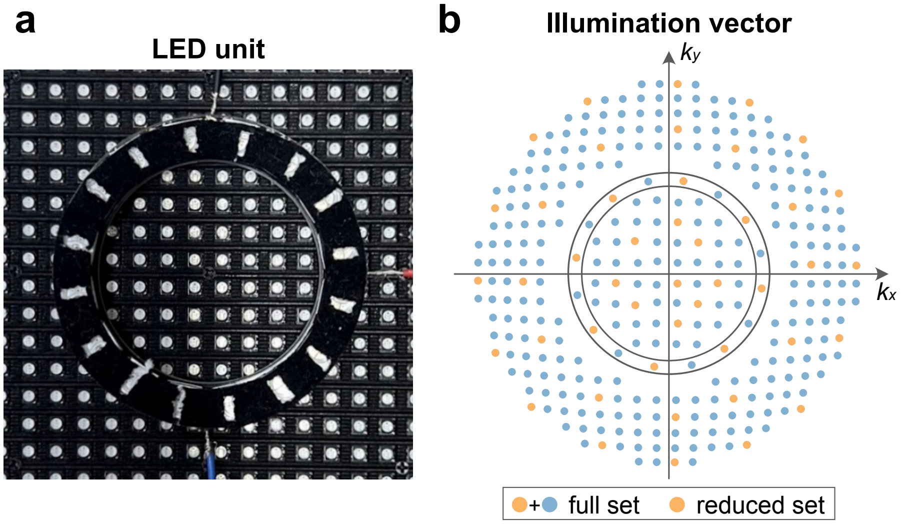

Figure S2: Arrangement of the LEDs in APIC. **a**, Image of the ring LED on top of a LED array. A black tape is covered on the ring LED to prevent stary light goes into the system due to the scattering of its white shell. The wires for the ring LED was glued in between the LED pairs on the LED array so that they do not block the LEDs. **b**, Illumination k vector for the full and reduced dataset. The ring LEDs sit in between the two black circles in the figure. We note the reduced dataset is a subset of the full set. The dots in orange shows the illuminations covered in the reduced dataset while the orange and blue together show the illuminations in the full dataset. To form the reduced set, the LEDs are chosen so that they are more uniformly distributed in k-space. k_x and k_y denote the spatial frequency coordinates.

To prevent the stray light entering the system, we covered the ring LED with a black tape. We note that there are some LEDs on the LED array being blocked by the ring attached onto it. These LEDs were not used in our experiment and thus we see a gap in the spatial frequency domain in Fig. S2b. We additionally note that such blocking has no obvious impact on the final reconstruction as the smallest overlap is still over 70% when these LED are dropped.

To construct the reduced dataset, we first uniformly sampled the illumination angle in the continuous spatial frequency space for the region corresponding to the brightfield, NA-matching, and darkfield measurements. For each of the sampled illumination angle (the ideal uniformly distributed illumination angle), we selected the LED with the smallest angle difference with respect to the desired one. By doing that, we made the LEDs distribute as uniformly as possible for our reduced dataset. The selected LEDs for the reduced dataset are shown in orange in Fig. S2b.

The reconstruction results using this reduced dataset are shown in Fig. S3. For comparison, we also included the reconstruction results when feeding in the entire dataset.

When FPM is not given the privilege of having a highly redundant dataset, its reconstruction result can be severely disturbed by the aberration of an imaging system whose phase variation exceeds $\frac{2\pi}{5}$ (NA of the objective: 0.25). We see that although FPM partially reconstructed the high spatial frequency information of the Siemens star target using the full dataset, it failed to maintain even the low spatial frequency information when a dataset with approximately 7 times fewer measurements was provided. In contrast, APIC, retrieved both the high and low spatial frequency information in either case. As we can see from Fig. S3, APIC

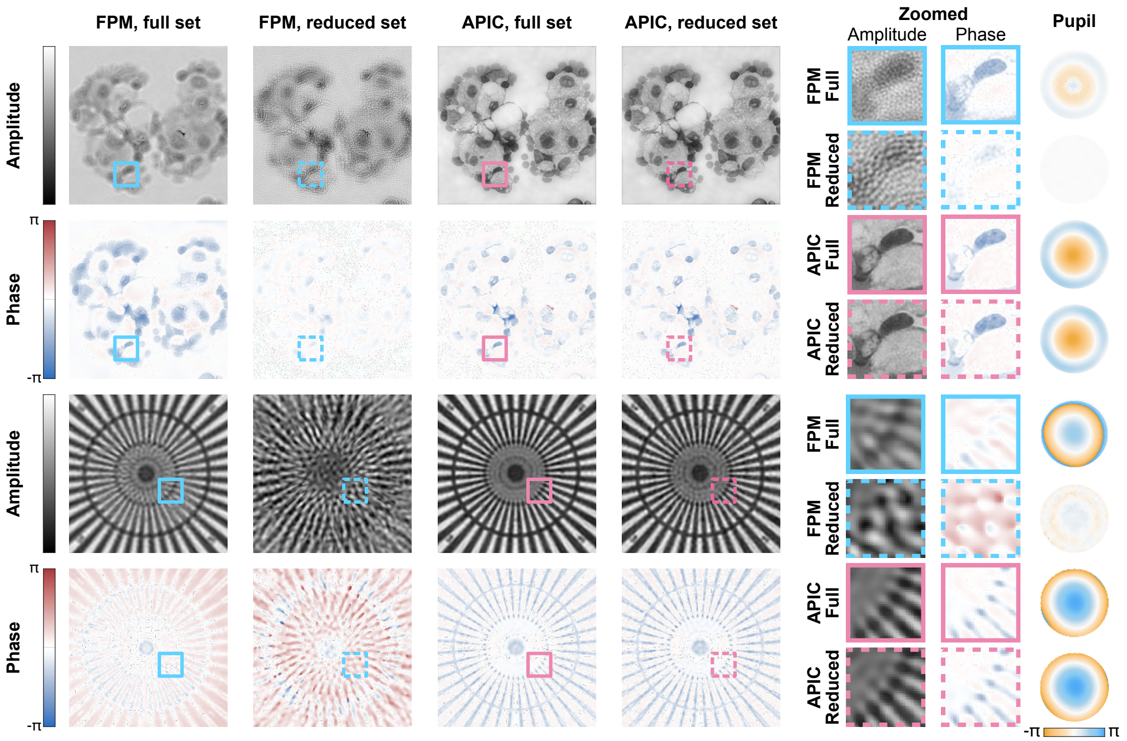

Figure S3: Reconstruction result of APIC and FPM using the full dataset and a reduced dataset. The full dataset is largely redundant as the overlap of two spectrum is around 87%. The nominal overlap ratio reduces to 65% for the reduced dataset. Siemens star target was defocused by 16 µm in this experiment. The zoomed images of the amplitude and phase reconstructions of APIC and FPM are shown on the right, respectively.

generated a reliable reconstructions using both the entire dataset and the reduced dataset.

3 Resolution quantification

To quantify the resolution achieved in APIC, we imaged a Siemens star target. In addition, we utilized LEDs whose illumination angles were smaller than the acceptance angle of our used objective for FPM. Those are the normal brightfield measurements required for FPM. We compared the resolution achieved by APIC and FPM. Here, we performed reconstruction on the in-focus Siemens star target shown in Fig. 4 of our main manuscript. To compare their resolution, we first calculated the smallest radius r where at least 10% contrast was preserved for any of the two reconstructed amplitude for the Siemens star target. We then plotted the radial profiles of APIC and FPM using this radius, respectively.

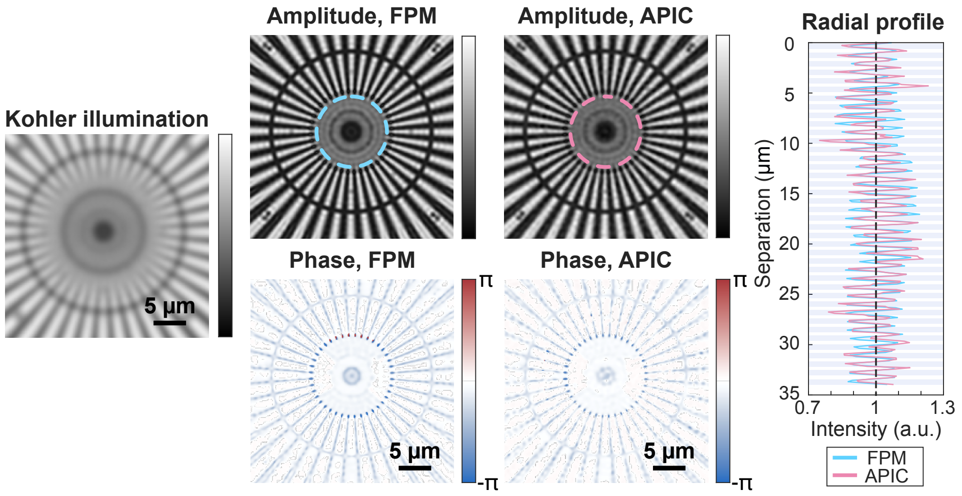

Figure S4: Quantify the resolution of FPM and APIC. We conducted the reconstruction using the in focus data and the reconstructed amplitude and phase of FPM and APIC are shown in the middle. Their radial profiles along the circles highlighted in the reconstructed amplitude images are shown on the right.

From Fig. S4, we see that APIC and FPM have similar radial profiles. The maximal illumination NA in this experiment is approximately 0.5 and the objective NA equals 0.25. We used red LEDs in the experiment and its central wavelength is 632 nm. Thus, the theoretical resolution of our system is 842 nm. Using the radial profiles in Fig. S4, the resolution we experimentally achieved for both FPM and APIC are approximately 867 nm, which is close to the theoretical resolution limit.

4 Reconstruction of a hematoxylin and eosin stained sample

In our main manuscript, the reconstruction result of the blue channel is shown in Fig. 5. Here, we showed the full comparison of reconstruction results of all three channels using FPM and APIC.

As we can see from Fig. S5, the reconstructions of FPM appeared grainy and the high spatial frequency information were only partially recovered. This indicates that FPM did not work well with this weakly absorptive sample. We also found that there were places where the sign of quantitative phase reconstructions of FPM for the green channel appeared to be flipped. In phase reconstruction shown in Fig. S5, some of the negative phases in the red and blue channel (phases shown in blue) become positive in the green channel (phases shown in red). The quantitative phases of APIC were much more consistent among all three channels. This indicates the aberration and complex field reconstruction of APIC is considerably more accurate compared with FPM.

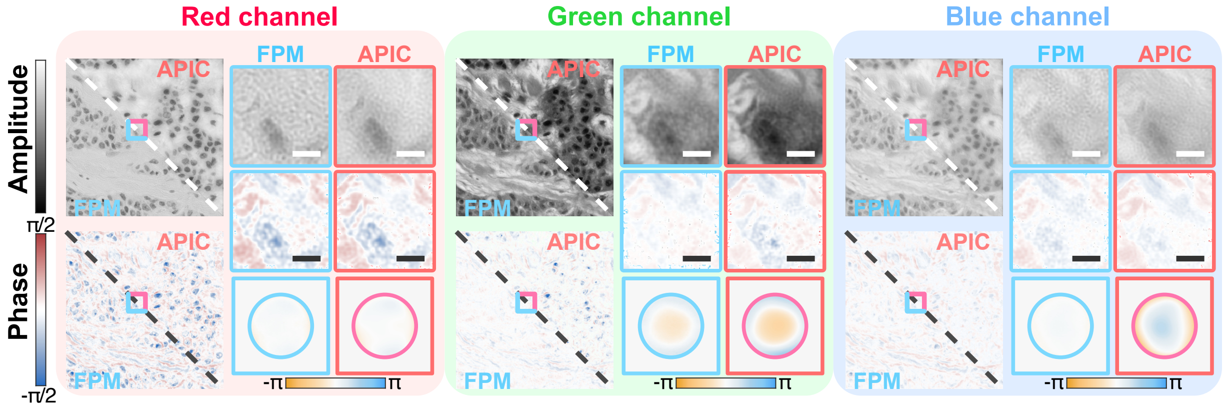

Figure S5: Reconstructed high-resolution image of hematoxylin and eosin (H&E) stained breast cancer cells. The complex field reconstructions and retrieved aberrations of each channel are shown in the red, green and blue highlighted boxes, respectively. For each group, FPM reconstructions are shown in the lower left and APIC reconstructions are shown in the upper right. The zoomed images of the amplitude and phase reconstructions are shown on the right of each boxes. Scale bar for the zoomed images: 5 μm.

5 Result of FPM and APIC when imaging a phase target

In this experiment, we made a standard phase sample to compare the reconstruction results of APIC and FPM. The sample we used in the experiment are polystyrene beads with a nominal diameter of 3 microns and the results are shown below in Fig. S6. From the results, we can see the phase profile from APIC is closer to a semi ellipse (reflecting the bead's profile) while the result of FPM shows a tiny peak on the top of the profile which makes it deviate from the elliptical profile. This observation matches up with our previous paper using Kramers-Kronig for phase reconstruction as both are analytical methods [2].

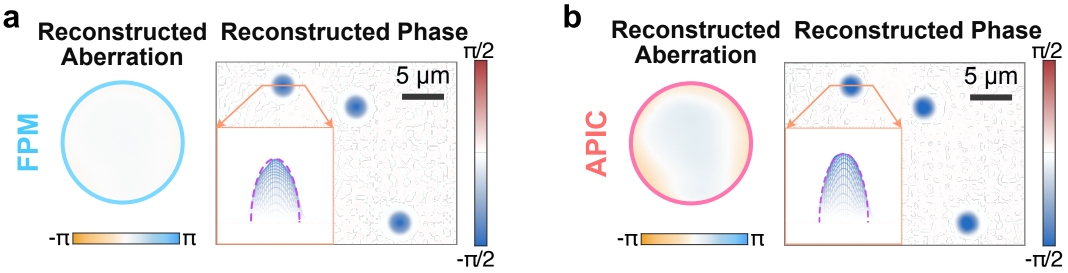

Figure S6: Experiment results using polystyrene beads (nominal diameter: 3 microns). **a**, Reconstructed complex field and aberration using FPM. **b**, Reconstructed complex field and aberration using APIC. The inset figures show the line profile of the phase reconstruction result. The purple dashed line in the inset of the phase reconstruction result indicates the ideal elliptical phase profile of a sphere.

6 Reconstruction time

In this section, we investigated the reconstruction time taken by FPM and APIC under different patch sizes. In this simulation, the overlap ratio was set to approximately 60%. The dataset consisted of 76 darkfield measurements and 16 NA-matching measurements. We used the same convergence criterion for first and second order FPM reconstruction methods as we did in experiments and simulations. We added moderate aberration in our simulation (the phase standard deviation of the simulated aberration is approximately 0.6 radian) so that FPM could give a reasonable reconstructions. We note that FPM might run much longer when dealing with more complicated cases, as the convergence criteria become more difficult to reach in the reconstruction of FPM. The reconstructions were conducted using a personal computer with 16 GB RAM (CPU: Intel Core i5-8259U).

Figure S7 shows the runtime comparison of FPM and AMIM as a function of the image patch size. When the side length of the patch equals 256, these three methods needs approximately the same amount of time to complete. We note that the reconstruction time of APIC also depends on the overlap ratio of the sampled spectrums. When using the highly aberrated images of the thyroid sample (Fig. 4 in our main manuscript), the reconstruction time of the second order FPM and APIC was comparable when reconstructing a square patch with side length of 512 pixels. Nonetheless, we see that APIC can be much faster when a small patch size is chosen. The computation efficiency advantage of APIC for small patch size is in line with another important computational consideration, namely parallel processing. By splitting the whole image into smaller patches, more processors can be simultaneously used for reconstruction.

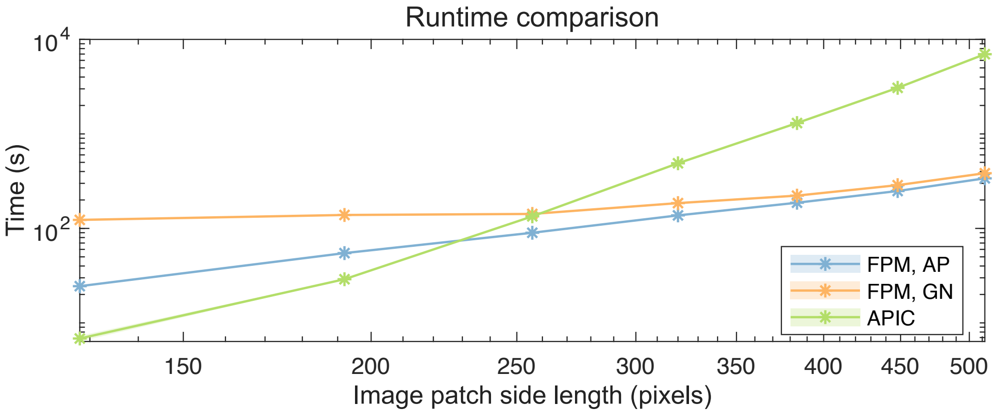

Figure S7: Runtime comparison of different algorithms. The patch sizes used in this simulation formed an arithmetic sequence ranging from 128 to 512 pixels, with a common difference of 64 pixels. The reconstruction of each method was repeated 5 times for each patch size, and the mean and standard deviation were calculated. The standard deviation is illustrated by the colored area behind the solid lines. In log-scale, the standard deviation is small compared with the mean value. Thus, we only see the solid lines showing up in the figure. AP denotes the original FPM reconstruction algorithm with EPRY and GN denotes the second order Gauss-Newton reconstruction algorithm.

7 Aberration correction

In this section, we conducted simulations under different aberration levels and used APIC to reconstruct the complex field. We simulated a complex USAF target as our object and added different aberrations in our measurements. We assumed the illumination angles were known and no noise was simulated. For the NA-matching angle illumination, 16 evenly spaced LEDs were simulated. The overlap ratio of two adjacent sampled spectrums is around 60%. The ground truth was generated such that its resolution was limited by the final synthetic NA in the simulation. To visually perceive different aberration levels, we also simulated the image that would be obtained by the aberrated imaging system under the normal incidence illumination. This normal incidence measurement was just for visualization and was not used in any APIC's reconstruction process.

The reconstruction results are shown in Fig. S8. From this simulation, we can see APIC is exceptionally tolerant against aberration and can accurately extract the aberration of the imaging system under extremely high aberration level (the largest phase standard deviation exceeds 1.5π). The reconstructed phase and amplitude for all cases matched up with the ground truth.

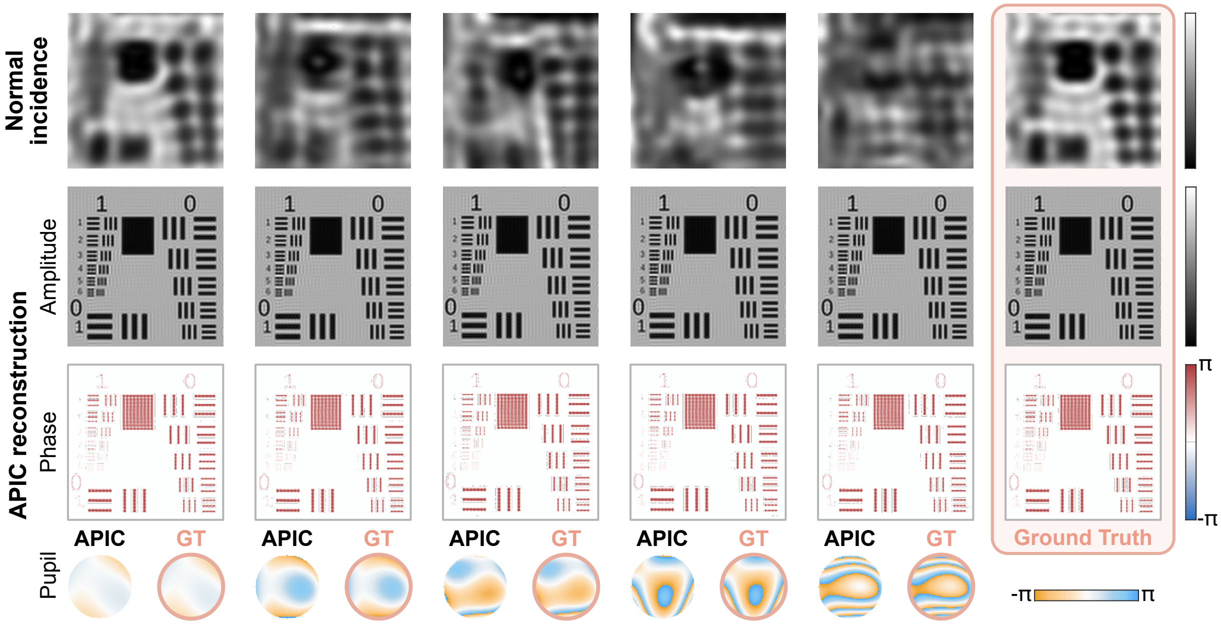

Figure S8: APIC under different levels of aberrations. The first row shows the corresponding image under normal incidence and is aberrated. The second and third rows are the reconstructed amplitude and phase, respectively. The last row is the reconstructed aberration and the actual simulated aberration. The last column shows the ground truth of object's amplitude and phase, as well as the image under normal incidence when captured by an aberration-free imaging system.

For comparison, we also used FPM to do reconstruction using two less aberrated cases above. We note that FPM needs a different set of brightfield measurements to complete the reconstruction. As such, we decided to match the number of brightfield measurements in FPM and APIC. If there were in total n_0 NA-matching measurements in APIC, we uniformly sampled n_0 brightfield illumination angles in our simulation. The darkfield measurements were shared by FPM and APIC. We adopted two different reconstruction algorithms for FPM, namely the original alternating projection algorithm (the original Gerchberg–Saxton algorithm combined with EPRY for aberration correction) [3–5] and the second order Gauss-Newton method [6, 7]. For FPM, we conducted the reconstruction with 6 different sets of parameters. As we knew the ground truth in our simulation, we manually chose one of these parameter sets so that its corresponding result is closest to the ground truth.

From the simulation results shown in Fig. S9, FPM worked well with mild aberrations. When the imaging system had a relatively small aberration (its phase standard deviation is approximately 0.15π), both the original alternating projection method (implemented with EPRY for aberration correction) and the second

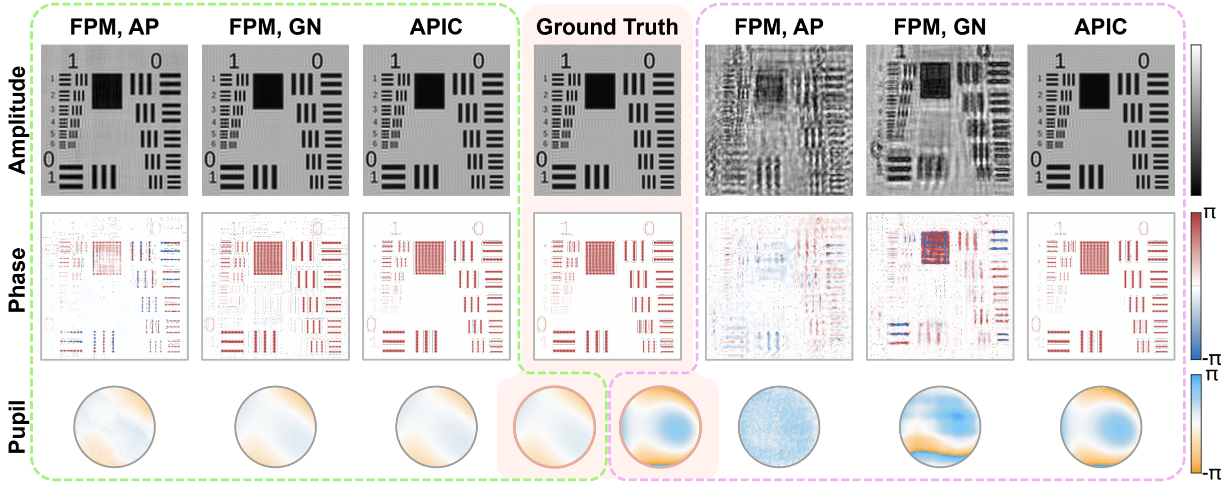

Figure S9: Comparison of FPM and APIC under moderate aberrations. AP denotes the alternating projection method (combined with EPRY) and GN denotes Gauss-Newton method. The ground truth of system's aberrations and the complex object are shown in the middle. In this simulation, two levels of moderate aberrations were applied. The results on the left correspond to the case where the gentler one among the two simulated aberration was used. The results on the right correspond to the more severe case.

order Gauss-Newton method successfully reconstructed the aberrations. Their amplitude reconstructions were also closely matched with the ground truth. However, we can see that the phase reconstruction of the second order method shows better correspondence to the ground truth. When the aberration in our simulation became a bit more severe (standard deviation reaches 0.4π), both FPM methods failed drastically. The second order FPM method works slightly better than the first order algorithm as it partially reconstructed the high-frequency information. However, those reconstructed images are severally distorted. APIC, on the contrary, works well with these different levels of aberrations.

8 Comparison under different signal-to-noise ratios

Here, we run APIC with different signal-to-noise ratios (SNRs) to see its performance under different scenarios. In this simulation, we assumed the illumination angles were known and an ideal aberration-free imaging system was used. Poisson noise was added in each measurement. The average number of photons for NA-matching measurements is shown on the top of Fig. S10. All other simulation parameters were the same.

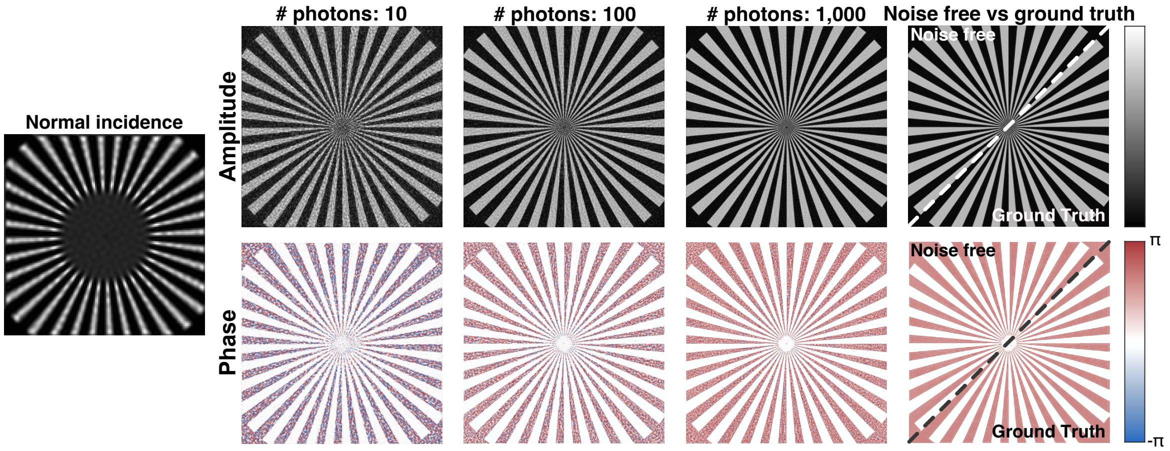

Figure S10: APIC under different SNRs. The measurement under normal incidence is shown on the far left, with no noise added. This measurement is for illustration purpose and is not used in APIC's reconstruction. Poisson noise was added to each measurement in this simulation. The numbers shown on the top are the average number of photons under NA-matching angle illumination. When the average photons is 10 for the NA-matching measurements, the average number of photons for all darkfield measurements is around 0.11.

If no noise was added to the measurement, we can see that APIC produced result that matched up with the ground truth. When the SNR was low, APIC became more noisy and exhibited degraded resolution. Nonetheless, it preserved the high frequency details that were not captured in the normal incidence measurement.

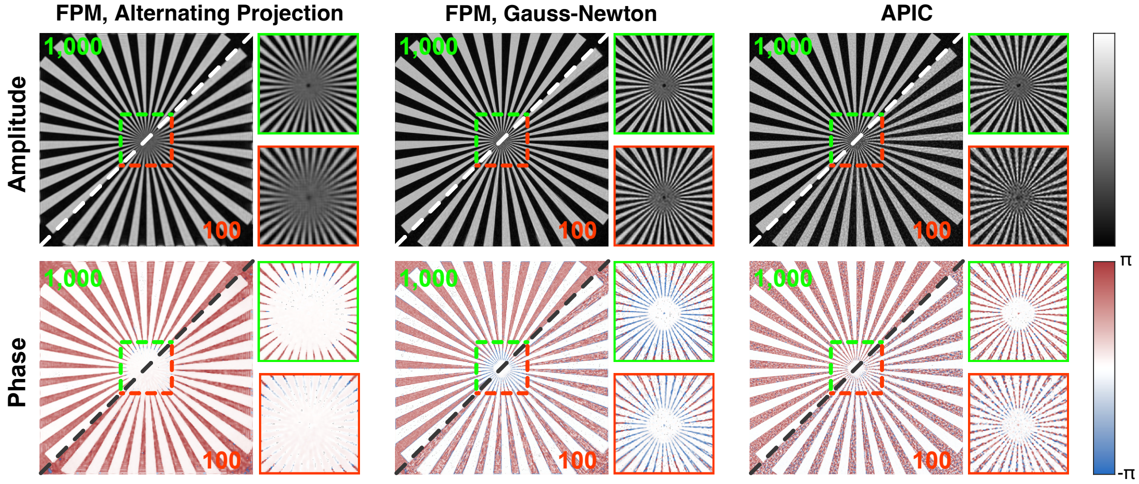

Figure S11: Comparison of FPM and APIC under different noise levels. For each group, the upper left panel shows the reconstruction result when the average number of photons of NA-matching measurements is 1,000, and the lower right panel shows the result when the number is 100. The zoomed version of the result within the box is shown on the far right of each group. For FPM, we performed the reconstruction using 6 different parameter sets and demonstrated the best reconstruction result we obtained.

Then we conducted another two simulations to compare APIC with FPM under different SNRs. As before, we replaced the NA-matching measurements with n_0 brightfield measurements to construct the dataset for FPM. All darkfield measurements were shared by APIC and FPM. For FPM, we chose 6 different sets of parameters and selected the best results in our simulation.

In the first simulation, we simulated a complex Siemens star target and added different levels of noise to the simulated dataset. We can see from Fig. S11 that the second order FPM algorithm performs well under low SNR (when the average number of photons equals 100 for the NA-matching measurements). This iterative algorithm trades the reconstruction speed for the additional tolerance of noise. The first order FPM reconstruction algorithm, which was much faster than the second order algorithm, performed worse than the other two in both cases. This is in accordance with previously reported results, which suggested the second order algorithm is much more robust than the first order alternating projection method [8].

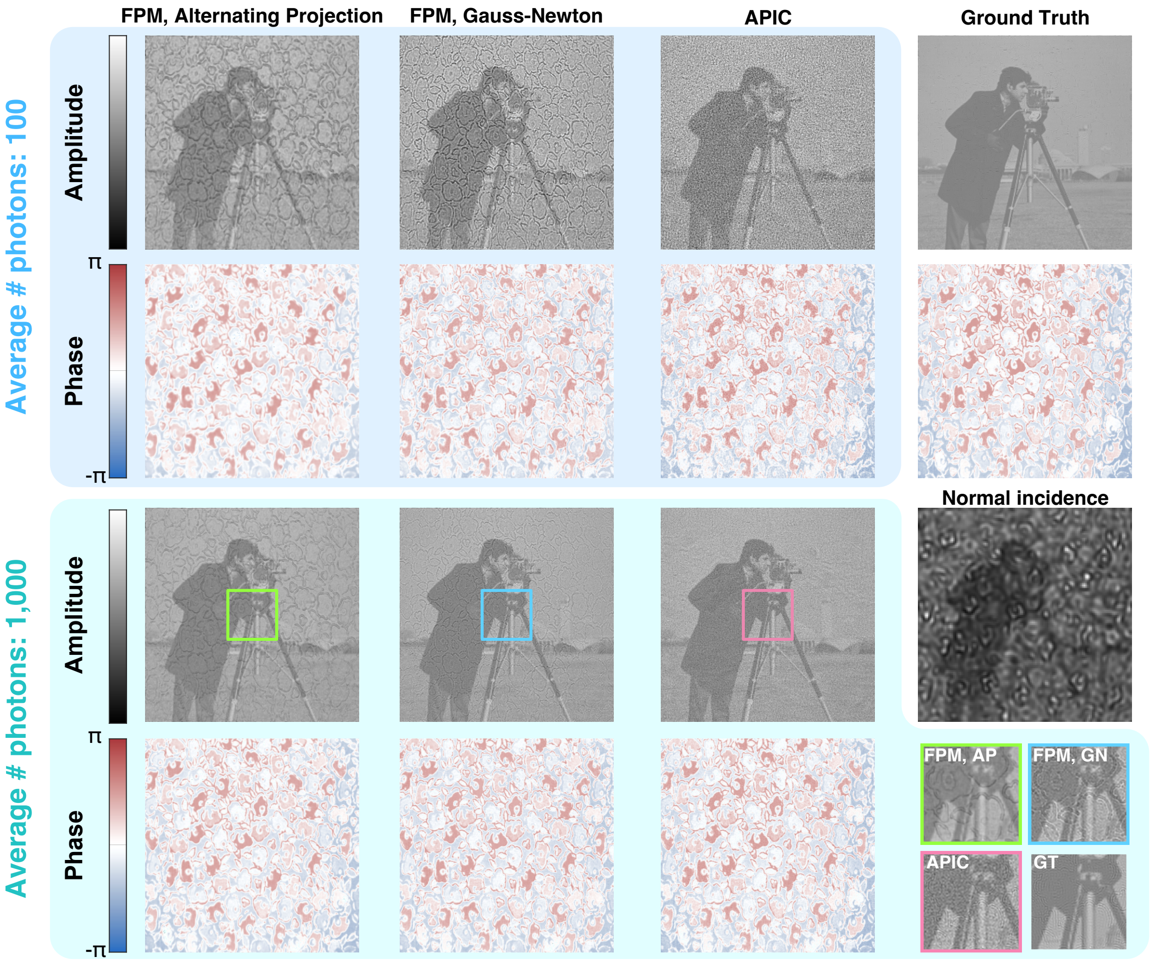

Figure S12: Reconstruction of a weak absorption target using FPM and APIC under different noise levels. The numbers shown on the left are the average number of photons under the matching angle. The zoomed version of images inside the highlighted boxes, along with the zoomed ground truth, are shown on the lower right of this figure. The image under normal incidence was simulated with a noise-free imaging system. For a complex sample whose amplitude and phase are different in morphology, cross-talk between the two is apparent in FPM's reconstruction. APIC, however, does not suffer from such severe cross-talk.

In our second simulation, we chose two different patterns for the amplitude and phase of the complex object. This complex object was designed to have a weak amplitude variation while preserving a relatively strong phase variation based on the common property for unstained biological samples. We see that there were severe cross-talks between phase and amplitude in both FPM algorithms using low SNR measurements. When SNR increased, such cross-talk became less prominent in FPM. Nonetheless, structures of the phases

remained visible in those cases. APIC, on the contrary, did not suffer from such cross-talk. Although the reconstruction of APIC with low SNR was noisy, it maintained the features of the ground truth amplitude and phase and showed almost no cross-talk between the two. Another thing we found in our simulation is that the reconstructed phase of APIC was closer to the ground truth. While the structural features of the reconstructed phase of FPM were similar to the ground truth, they quantitatively differed from each other. For the low SNR dataset, the range of the reconstructed phase of FPM appeared to be compressed. This indicates that if the reconstruction artifact is undesired and the fidelity is of concern, APIC is definitely a preferable choice when imaging most unstained biological samples, even under low SNR conditions.

9 Number of NA-matching measurements required in APIC

In this section, we simulated different numbers of NA-matching measurements to see the required measurements for APIC to accurately reconstruct the imaging system's aberration. In our simulation, we only introduced aberration to the imaging system and all other parameters were assumed to be ideal. We assumed the illumination orientations were azimuthally uniformly distributed, which means their corresponding LEDs were uniformly distributed along a certain ring. The reconstructed aberrations are shown in Fig. S13.

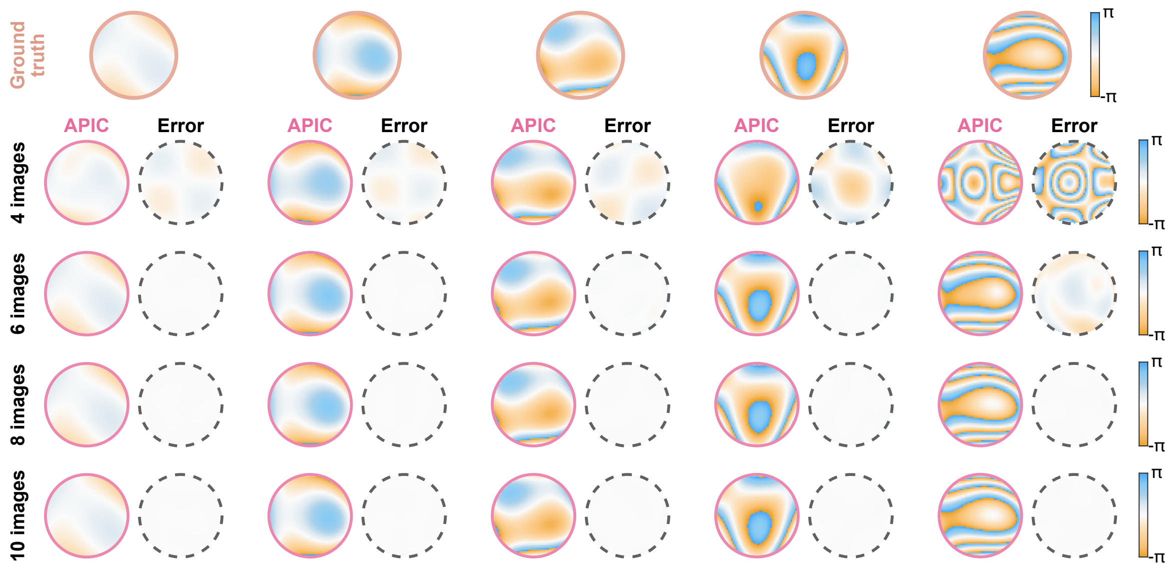

Figure S13: Reconstructed aberration of APIC using different numbers of NA-matching measurements. The ground truths in the simulation are shown on top of each group. The number of NA-matching measurements used in the simulation are dented on the far left size of the image. In addition to the reconstructed aberration, we also included the error map between this reconstructed aberration and the ground truth. This error map is placed next to the APIC's reconstruction and is highlighted by a black dashed circle.

When using 4 images, APIC did not obtain a good aberration estimate. However, we can see that when there were 6 NA-matching measurements, APIC successfully reconstructed mild to moderately high aberrations (their phase standard deviation is below 0.8π). However, residual aberration exists in the reconstructed aberration under severe aberrations (phase standard deviation exceeds 1.5π), as depicted in the corresponding error map in Fig. S13. When the number of NA-matching measurements increased to 8, we see that there are no residual aberrations in APIC's reconstructions regardless of the severeness of the aberration. In general, 6 to 8 NA-matching measurements would be sufficient for generating an accurate aberration estimation in most cases.

10 Inaccurate illumination angle estimates

It is previously shown that inaccurate illumination angle results in degradation of reconstruction using Kramers-Kronig method [2]. Here, we simulated errors in the angle calibration to see how APIC performs under different levels of calibration error. Random uniformly distributed estimation error was introduced to the actual illumination angles. The maximal amount of error in our simulation was proportional to the maximal acceptance angle of the imaging system. As the illumination angle was converted to the spatial frequency vector in practice, this maximum error is denoted by the ratio of the error to the k-vector of the maximal acceptance angle. In our simulation, we assumed the estimate of the NA-matching angle was no larger than the acceptance angle of the imaging system.

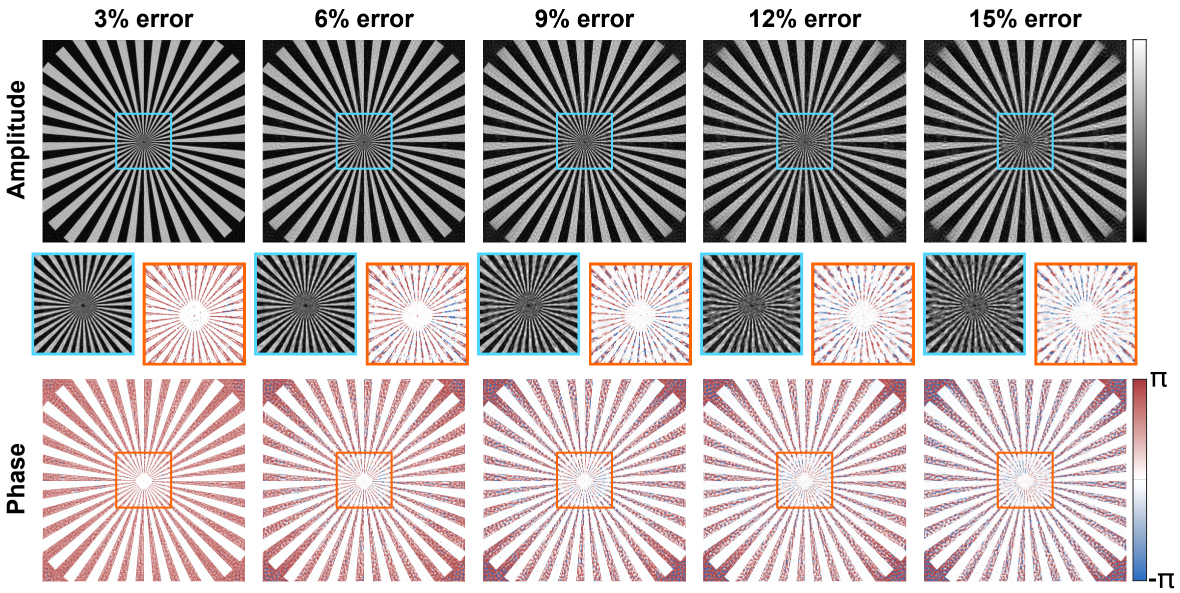

Figure S14: APIC reconstruction with different levels of angle error. In this simulation, we added randomly generated angle errors to the actual illumination angles and fed this inaccurate angle estimate to APIC when performing the reconstruction. The numbers on top of each group are the maximal possible error in our simulation. If the maximal possible error is denoted by γ, the simulated angle errors for both the horizontal and vertical direction were uniformly sampled in $[-\gamma, \gamma]$. For each NA-matching measurement, we forced the corresponding angle estimate to be smaller than the acceptance angle of the system by tuning the sign of this randomly generated error.

From the simulation results, we can see that APIC performance was well-maintained until the error reached 9%. Beyond that, the reconstructions showed obvious artifacts. This indicates that the alignment requirement of our proposed APIC is fairly relaxed. In other words, APIC is tolerant to the LED position errors to a good extent.

11 Result of spatial-domain Kramers-Kronig method and APIC

Here, we reconstructed the complex field using the recently proposed algorithm, which does not take complex aberration into consideration [2, 9, 10]. We used a USAF target for simulation. The LED illumination angles were assumed to be known and an aberration-free imaging system was used in our simulation. As the spatial Kramers-Kronig method cannot perform reconstruction with darkfield measurements, its resolution is limited. APIC is the first of its kind that can do analytical complex field reconstruction using darkfield measurements. With the more general phase retrieval framework established in APIC, we can see that the resolution of APIC is much enhanced compared with the spatial Kramers-Kronig method.

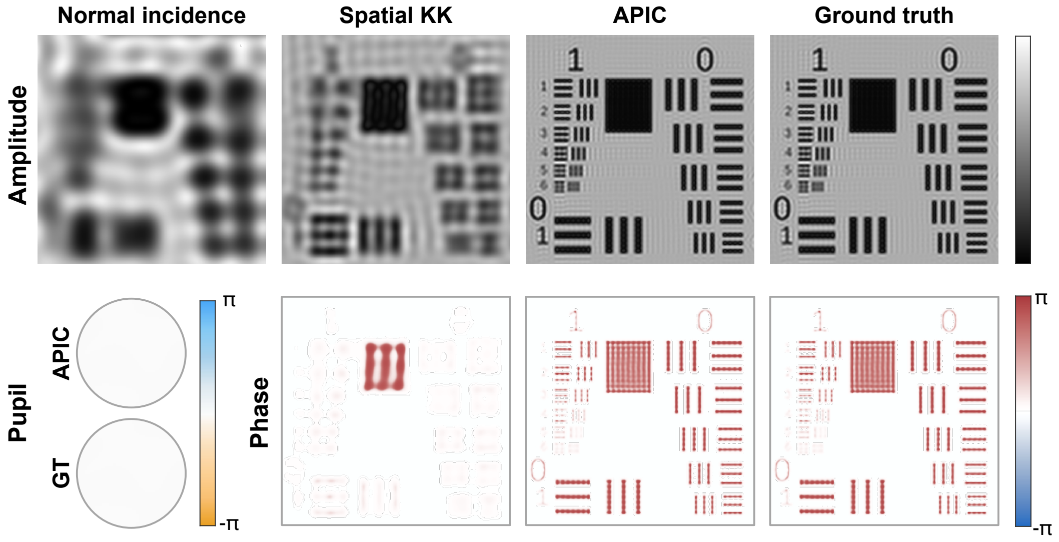

Figure S15: Comparison of the spatial Kramers-Kronig (spatial KK) method and APIC. The image under normal incidence is shown on the left and the ground truth of the amplitude and phase of the USAF target we used in the simulation are shown on the right. The ground truth of the aberration and APIC recovered aberration are shown on the lower left of this image. In this simulation, no aberration was introduced. GT: ground truth.

12 Derivation of APIC

In this section, we present the mathematical form of the forward model and reconstruction step of APIC. We begin by defining the forward model and introducing the notations in our derivation. Subsequently, we present the mathematical derivation of APIC, which involves three main steps: reconstructing the field using NA-matching measurements (measurements acquired under tilted illumination whose angle matches with the maximal acceptance angle of an imaging system), extracting and correcting aberrations, and reconstructing the complex field using darkfield measurements.

In our derivation, we first present the Kramers-Kronig method, which enables the reconstruction of complex fields from NA-matching measurements [2, 9–11]. Using these reconstructed fields, we analytically retrieve the aberration of the imaging system. The extracted aberration is then applied to correct the currently reconstructed aberrated fields and the subsequent darkfield associated field reconstructions. The aberration-corrected reconstructed complex field serves as our initial *a priori* knowledge, referred to as the known field, and its Fourier transform as the known spectrum. To achieve high-resolution imaging, we incorporate darkfield measurements and use them to expand the reconstructed sample spectrum in an orderly way. At each sub-step, we reconstruct the spectrum corresponding to an unused darkfield measurement whose illumination angle is the smallest among all remaining unused measurements. This newly reconstructed spectrum, together with the original reconstructed spectrum, serves as the new *a priori* knowledge in the subsequent reconstructions. To recover the field associated with one darkfield measurement, we focus on the spatial frequency space and form a linear equation with respect to the unknown spectrum. By solving this equation, we obtain a closed-form solution of the unknown spectrum sampled in this darkfield measurement. Adding this newly reconstructed spectrum effectively expands the sample spectrum coverage to achieve higher resolution.

After all measurements are reconstructed, we obtain a high-resolution, aberration-free complex field reconstruction in a purely analytical manner.

12.1 The forward model

When a thin sample is illuminated by a plane wave emitted by the ith ($i = 1, 2, \cdots, n$) LED with a transverse k-vector $\mathbf{k}_i$ and then imaged by an optical system, the modulated sample spectrum is given by

$$\hat{S}_i(\mathbf{k}) = \hat{O}(\mathbf{k} - \mathbf{k}_i)H(\mathbf{k}), \tag{1}$$

where H is the coherent transfer function (CTF) associated with the imaging system, $\hat{O}$ is the original sample's spectrum, $\hat{S}_i$ is the ith sampled spectrum, and $\mathbf{k}$ is the 2D (transverse) spatial frequency vector. Reciprocally, we denote the spatial coordinate on the sample plane with $\mathbf{x}$. Without loss of generality, we assume $\hat{O}(\mathbf{0})$ is a real number, as the absolute average phase delay is not our interest. For a thin sample, we can further assume that the majority of the incident illumination light does not change its original direction. That is, at any position $\mathbf{x}$ we have

$$\left|\left(\mathcal{F}^{-1}\big[\hat{O}(\mathbf{k})\delta(\mathbf{k})\big]\right)(\mathbf{x})\right| > \left|\left[\mathcal{F}^{-1}\Big(\hat{O}(\mathbf{k})\big[1-\delta(\mathbf{k})\big]\Big)\right](\mathbf{x})\right|, \tag{2}$$

where $\mathcal{F}^{-1}$ is the inverse Fourier transform, $\big[\mathcal{F}^{-1}(\cdot)\big](\mathbf{x})$ means the inverse Fourier transform is evaluated at $\mathbf{x}$, $|\cdot|$ gives the modulus of a complex number, and $\delta(\mathbf{k})$ is the "Kronecker delta" which is defined by

$$\delta(\mathbf{k}) = \begin{cases} 1, & \mathbf{k} = \mathbf{0}, \\ 0, & \text{otherwise.} \end{cases} \tag{3}$$

We note that $\delta(\mathbf{k})$ is not a proper definition of a Kronecker delta function which is defined over integers. To be rigorous, one needs to define a function equals one in an ε-neighbor around the origin and zero otherwise. Then, we can proceed to get the following result by approaching to an infinitesimal neighbor. Here, we simplify it so that the notation is more approachable. The physical meaning of this assumption is that the ballistic light exiting from the sample plane is dominant over the scattering light everywhere within the field of view.

As only the intensity of the light field is directly measured, the signal we get from a camera is

$$I_i(\mathbf{x}) = \left|\big[\mathcal{F}^{-1}(\hat{S}_i)\big](\mathbf{x})\right|^2 = \big|S_i(\mathbf{x})\big|^2, \tag{4}$$

where I_i is the ith image captured when lighting up the ith LED, and S_i denotes the ith sampled field in real space (the inverse Fourier transform of $\hat{S}_i$).

It is worth noting that this intensity measurement is insensitive to phase shift applied to the inverse Fourier transform

$$\left|\left[\mathcal{F}^{-1}(\hat{S}_i)\right](\mathbf{x})e^{j\xi(\mathbf{x})}\right|^2 = \left|\left[\mathcal{F}^{-1}(\hat{S}_i)\right](\mathbf{x})\right|^2 = I_i(\mathbf{x}), \tag{5}$$

where $\xi(\mathbf{x})$ stands for an arbitrary phase function and j is the unit imaginary number. Thus, we can choose a particular phase ramp, namely $-2\pi\mathbf{k}_i \cdot \mathbf{x}$, which effectively shifts $\hat{S}_i$ along the opposite direction of $\mathbf{k}_i$. Using the properties of Fourier transform when applying this special phase ramp, the measured intensity image is identical to the intensity of the inverse Fourier transform of the following (translated) spectrum

$$\hat{S}'_i(\mathbf{k}) = \left[\mathcal{F}(S'_i)\right](\mathbf{k}) = \left[\hat{S}_i * \mathcal{F}(e^{-j2\pi\mathbf{k}_i\cdot\mathbf{x}})\right](\mathbf{k}) = \hat{O}(\mathbf{k})H(\mathbf{k}+\mathbf{k}_i), \tag{6}$$

where $\cdot$ is the dot product, $S'_i(\mathbf{x}) \coloneqq S_i(\mathbf{x})e^{-2\pi j\mathbf{k}_i\cdot\mathbf{x}}$, and $*$ is convolution. This model suggests that different components of sample's spectrum can be measured with different illumination angles. By increasing the illumination angle, we can effectively sample high spatial frequency information which is normally inaccessible due to limited numeric aperture (NA) of an imaging system.

The CTF support of an imaging system can be reasonably assumed as circular with a NA-dependent radius k_{NA}, and its phase ϕ of the CTF function fully depicts the system's aberrations. For simplicity, we abuse the notation and use NA to represent this radius k_{NA} in the following section. We then have (note NA means k_{NA})

$$\begin{aligned} H(\mathbf{k}) &= \mathrm{Circ}_{\mathrm{NA}}(\mathbf{k})e^{j\phi(\mathbf{k})} = \mathbb{1}(|\mathbf{k}| \le \mathrm{NA})e^{j\phi(\mathbf{k})} \\ &= \begin{cases} e^{j\phi(\mathbf{k})}, & \text{if } |\mathbf{k}| \le \mathrm{NA}, \\ 0, & \text{otherwise}, \end{cases} \end{aligned} \tag{7}$$

where $\phi(\mathbf{k})$ stands for the system's aberration, and $\mathrm{Circ}_{\mathrm{NA}}(\mathbf{k}) \coloneqq \mathbb{1}(|\mathbf{k}| \le \mathrm{NA})$ is an indicator function that gives one when the modulus of the k-vector is below the system NA and zero otherwise.

We further decompose $\hat{O}(\mathbf{k})$ to its amplitude and phase. Together with Eq. 7, we can rewrite Eq. 6, which gives

$$\hat{S}'_i(\mathbf{k}) = \hat{O}(\mathbf{k})H(\mathbf{k}+\mathbf{k}_i) = \hat{A}(\mathbf{k})e^{j\hat{\alpha}(\mathbf{k})}\,\mathrm{Circ}_{\mathrm{NA}}(\mathbf{k}+\mathbf{k}_i)e^{j\phi(\mathbf{k}+\mathbf{k}_i)}, \tag{8}$$

where $\hat{A}(\mathbf{k}) \coloneqq \left|\hat{O}(\mathbf{k})\right| \in \mathbb{R}$ is the amplitude of the sample's spectrum and $\hat{\alpha}(\mathbf{k}) \coloneqq \arg\left[\hat{O}(\mathbf{k})\right] \in \mathbb{R}$ is its phase (the operator arg gives the argument of a complex number).

In the following two subsections, we proceed with Eq. 6 and show that the closed-form solution of the sample's spectrum $\hat{O}(\mathbf{k})$ can be obtained (sections 12.2 and 12.4), and the aberration of the imaging system $\phi(\mathbf{k})$ can also be analytically retrieved (section 12.3). This is achieved by only using the NA-matching and darkfield measurements. For simplicity, in the following discussion, we assume all $|\mathbf{k}_i| \ge \mathrm{NA}$ and $\mathbf{k}_i$ is ordered so that $|\mathbf{k}_i| \le |\mathbf{k}_{i+1}|$.

12.2 Reconstruction under the NA-matching angle illumination

For integrity, we reproduce the main result of recent works on spatial Kramers-Kronig relations which enables complex field reconstruction when the illumination angle exactly matches with the maximal acceptance angle of the objective [2, 9, 10]. However, the aberration consideration is missing in these existing works. Here, we take the imaging system's aberration into consideration. The main result can be found in Eqs. 32 and 33. In this subsection, we only consider the first n_0 measurements whose k-vector $\mathbf{k}_i$ satisfies

$$|\mathbf{k}_i| = \mathrm{NA}, \quad i = 1, 2, \cdots, n_0, \quad \text{where } n_0 \coloneqq \max\left\{i \in \mathbb{N} \,\middle|\, |\mathbf{k}_i| = \mathrm{NA}\right\}. \tag{9}$$

We call these measurements as the NA-matching measurements. Instead of presenting this result using directional Hilbert transform and 1D Kramers-Kronig relations, we take the opportunity to put more focus on the underlying structure of the (logarithm mapped) signal's Fourier transform. To be specific, we will construct a signal whose Fourier transform is one-sided (which will be defined later), and show that this allows us to calculate the imaginary part of the signal from its real part based on the fact that Fourier transform is a linear operator. This carefully constructed signal can be mapped back to the complex field we want. We note that the approach presented here can also be generalized to higher dimensional spaces in an

intuitive way. Although this method allows analytical complex field reconstruction, we found that both our desired sample's field and the aberration function of the imaging system are entangled in the reconstructed field.

As our goal is computing the imaginary part of a complex signal from its real part using Kramers-Kronig relations, the real part must be known first. However, we only measure the intensity (squared modulus) of a complex field, which is neither its real or imaginary part. To solve this mismatch, we apply a nonlinear transformation that maps the intensity and phase of a complex number to the real and imaginary part of its output, respectively. This can be done by taking the logarithm of a nonzero complex number. Applying this to the complex field $S_i'(\mathbf{x})$ in a point-wise manner, we have

$$\log\left[S_i'(\mathbf{x})\right] = \log\left[\left|S_i'(\mathbf{x})\right|\right] + j\arg\left[S_i'(\mathbf{x})\right]. \tag{10}$$

The nonzero condition is guaranteed by Eq. 2. Note that the first term on the right-hand side is purely real and the second term is purely imaginary. As the intensity $I_i(\mathbf{x}) = \left|S_i(\mathbf{x})\right|^2 = \left|S_i(\mathbf{x})\cdot e^{-2\pi j\mathbf{k}_i\cdot\mathbf{x}}\right|^2 = \left|S_i'(\mathbf{x})\right|^2$ is measured, we know everything about the real part of $\log\left[S_i'(\mathbf{x})\right]$. What remains is to verify that its imaginary part can be reconstructed using this known real part.

In the remaining part of this section, we will show that the Fourier transform of this transformed signal is one-sided. Such structure allows us to perform our desired reconstruction. We first give the definition of a signal being one sided.

Definition 12.2.1. *For any positive integer $m \in \mathbb{Z}^+$, we say $g : \mathbb{R}^m \to \mathbb{C}$ is one sided if there exists a nonzero vector $\mathbf{e} \in \mathbb{R}^m, \mathbf{e} \neq \mathbf{0}$ such that its Fourier transform $\hat{g}$ satisfies*

$$\hat{g}(\mathbf{k}) = 0, \quad \forall \mathbf{k} \in \mathbb{R}^m \ s.t.\ \mathbf{k}\cdot\mathbf{e} < 0. \tag{11}$$

We say g is strictly one sided if $\hat{g}(\mathbf{k}) = 0,\ \forall \mathbf{k} \in \mathbb{R}^m\ s.t.\ \mathbf{k}\cdot\mathbf{e} \leq 0$.

To show the transform signal is one sided, we are going to factor out the "offset" field, $\mathcal{R}(\mathbf{x})$, which is the field that does not change its direction

$$\mathcal{R}(\mathbf{x}) = \Big(\mathcal{F}^{-1}\big[\hat{S}_i'(\mathbf{k})\delta(\mathbf{k})\big]\Big)(\mathbf{x}) = \Big(\mathcal{F}^{-1}\big[\hat{O}(\mathbf{k})H(\mathbf{k}+\mathbf{k}_i)\delta(\mathbf{k})\big]\Big)(\mathbf{x}) = re^{j\phi(\mathbf{k}_i)}, \tag{12}$$

where r is real. When we factor out this field from $S_i'(\mathbf{x})$, we have

$$\frac{S_i'(\mathbf{x})}{\mathcal{R}(\mathbf{x})} = \frac{S_i'(\mathbf{x})}{re^{j\phi(\mathbf{k}_i)}} = \frac{S_i'(\mathbf{x})e^{-j\phi(\mathbf{k}_i)}}{r}. \tag{13}$$

We can see that $\phi(\mathbf{k}_i)$ serves as a phase offset and r can be treated as a normalization factor. Note that r and $\phi(\mathbf{k}_i)$ are unknown in reality, and only the intensity of $S_i(\mathbf{x})$ is measured. Thus, we primarily focus on $S_i'(\mathbf{x})e^{-j\phi(\mathbf{k}_i)}$ in our following derivation because adding a phase offset to a complex field does not change its intensity.

Applying the logarithm transformation to the offset field $S_i'(\mathbf{x})e^{-j\phi(\mathbf{k}_i)}$, we have

$$\log\left[S_i'(\mathbf{x})e^{-j\phi(\mathbf{k}_i)}\right] = \log\left[\frac{S_i'(\mathbf{x})e^{-j\phi(\mathbf{k}_i)}}{r}\right] + \log(r) = \log\left[1 + \frac{S_i'(\mathbf{x})e^{-j\phi(\mathbf{k}_i)} - r}{r}\right] + \log(r). \tag{14}$$

With the assumption that the majority of the light does not change its direction (Eq. 2), we have the offset field is larger than the sample modulated field $r = \left|\mathcal{R}(\mathbf{x})\right| > \left|S_i'(\mathbf{x}) - \mathcal{R}(\mathbf{x})\right| = \left|S_i'(\mathbf{x}) - re^{j\phi(\mathbf{k}_i)}\right|$. So, we have $r > \left|S_i'(\mathbf{x})e^{-j\phi(\mathbf{k}_i)} - r\right|\left|e^{j\phi(\mathbf{k}_i)}\right| = \left|S_i'(\mathbf{x})e^{-j\phi(\mathbf{k}_i)} - r\right|$. Thus, we can write the first term on the right-hand side in a convergent Taylor series (we will see this makes it easier to analyze the structure of its Fourier transform)

$$T(\mathbf{x}) \coloneqq \log\left[1 + \frac{S_i'(\mathbf{x})e^{-j\phi(\mathbf{k}_i)} - r}{r}\right] = \sum_{m=1}^{\infty}\frac{(-1)^{m+1}}{m}\frac{\left[S_i'(\mathbf{x})e^{-j\phi(\mathbf{k}_i)} - r\right]^m}{r^m}. \tag{15}$$

Note this applies to all $\mathbf{x}$. For simplicity, we define $\Delta_i(\mathbf{x})$ as

$$\Delta_i(\mathbf{x}) \coloneqq S_i'(\mathbf{x})e^{-j\phi(\mathbf{k}_i)} - r = \left[S_i'(\mathbf{x}) - re^{j\phi(\mathbf{k}_i)}\right]e^{-j\phi(\mathbf{k}_i)} = \left[S_i'(\mathbf{x}) - \mathcal{R}(\mathbf{x})\right]e^{-j\phi(\mathbf{k}_i)}, \tag{16}$$

and Eq. 14 can be rewritten as

$$\log\left[S_i'(\mathbf{x})e^{-j\phi(\mathbf{k}_i)}\right] = \log(r) + T(\mathbf{x}) = \log(r) + \sum_{m=1}^{\infty} \frac{(-1)^{m+1}}{m}\frac{\Delta_i^m(\mathbf{x})}{r^m}. \tag{17}$$

The Fourier transform of $\Delta_i(\mathbf{x})$ yields

$$\hat{\Delta}_i(\mathbf{k}) = \big[\mathcal{F}(\Delta_i)\big](\mathbf{k}) = \Big[\mathcal{F}\Big(\big[S_i'(\mathbf{x}) - \mathcal{R}(\mathbf{x})\big]e^{-j\phi(\mathbf{k}_i)}\Big)\Big](\mathbf{k}) = \hat{S}_i'(\mathbf{k})\big[1-\delta(\mathbf{k})\big]e^{-j\phi(\mathbf{k}_i)}. \tag{18}$$

Lemma 12.2.1. *For $\mathbf{x} \in \mathbb{R}^2$, let $f_1(\mathbf{x}) : \mathbb{R}^2 \to \mathbb{C}$ and $f_2(\mathbf{x}) : \mathbb{R}^2 \to \mathbb{C}$ be two complex l_2 function with Fourier transform $\hat{f}_1(\mathbf{k})$ and $\hat{f}_2(\mathbf{k})$, respectively. Assume there exists a (common) nonzero vector $\mathbf{e} \in \mathbb{R}^2$, $\mathbf{e} \neq \mathbf{0}$ such that*

$$\hat{f}_1(\mathbf{k}) = \hat{f}_2(\mathbf{k}) = 0, \forall \mathbf{k} \text{ s.t. } \mathbf{k}\cdot\mathbf{e} < 0, \tag{19}$$

then their product $f'(\mathbf{x}) = f_1(\mathbf{x})f_2(\mathbf{x})$ is one sided. Furthermore, if f_1 and f_2 are strictly one sided (with the same $\mathbf{e}$), their product is also strictly one sided.

Proof: We take the Fourier transform on both sides, which yields

$$\hat{f}'(\mathbf{k}) = \big[\mathcal{F}(f')\big](\mathbf{k}) = \big[\mathcal{F}(f_1 f_2)\big](\mathbf{k}) = \Big(\big[\mathcal{F}(f_1)\big] * \big[\mathcal{F}(f_1)\big]\Big)(\mathbf{k}) = \big[\hat{f}_1 * \hat{f}_2\big](\mathbf{k}). \tag{20}$$

For $\mathbf{k}_0 \in \mathbb{R}^2$ such that $\mathbf{k}_0 \cdot \mathbf{e} < 0$ ($\mathbf{e} \neq \mathbf{0}$), we have

$$\begin{aligned}\hat{f}'(\mathbf{k}_0) = \big[\hat{f}_1 * \hat{f}_2\big](\mathbf{k}_0) &= \int d\mathbf{k}'\hat{f}_1(\mathbf{k}')\hat{f}_2(\mathbf{k}_0-\mathbf{k}') \\ &= \int_{\mathbf{k}'\cdot\mathbf{e}<0} d\mathbf{k}'\hat{f}_1(\mathbf{k}')\hat{f}_2(\mathbf{k}_0-\mathbf{k}') + \int_{\mathbf{k}'\cdot\mathbf{e}\geq 0} d\mathbf{k}'\hat{f}_1(\mathbf{k}')\hat{f}_2(\mathbf{k}_0-\mathbf{k}').\end{aligned} \tag{21}$$

If $\mathbf{k}'\cdot\mathbf{e} < 0$, we have $\hat{f}_1(\mathbf{k}') = 0$. If $\mathbf{k}'\cdot\mathbf{e} \geq 0$, we have $(\mathbf{k}_0 - \mathbf{k}')\cdot\mathbf{e} = \mathbf{k}_0\cdot\mathbf{e} - \mathbf{k}'\cdot\mathbf{e} < 0$. As f_1 and f_2 are both one sided and share the same $\mathbf{e}$, we can conclude

$$\hat{f}'(\mathbf{k}_0) = \int_{\mathbf{k}'\cdot\mathbf{e}<0} d\mathbf{k}'\hat{f}_1(\mathbf{k}')\hat{f}_2(\mathbf{k}_0-\mathbf{k}') + \int_{\mathbf{k}'\cdot\mathbf{e}\geq 0} d\mathbf{k}'\hat{f}_1(\mathbf{k}')\hat{f}_2(\mathbf{k}_0-\mathbf{k}') = 0, \quad \forall \mathbf{k}_0 \text{ s.t. } \mathbf{k}_0\cdot\mathbf{e} < 0. \tag{22}$$

That is, f' is one sided. The proof for f_1 and f_2 being strictly one sided follows the same structure. To see that, we calculate the integration for $\mathbf{k}'\cdot\mathbf{e} \leq 0$ and $\mathbf{k}'\cdot\mathbf{e} > 0$. Then, we can prove the strict version with the same technique. □

We can easily see that $\Delta_i(\mathbf{x})$ is one sided. To show this, we choose $\mathbf{e} = -\mathbf{k}_i$. As the illumination angle is matched with NA (Eq. 9), we can verify that for arbitrary $\mathbf{k}$ such that $\mathbf{k}\cdot\mathbf{e} < 0$, we have

$$|\mathbf{k}+\mathbf{k}_i| = \sqrt{|\mathbf{k}|^2 + |\mathbf{k}_i|^2 + 2\mathbf{k}\cdot\mathbf{k}_i} = \sqrt{|\mathbf{k}|^2 + |\mathbf{k}_i|^2 - 2\mathbf{k}\cdot\mathbf{e}} > \sqrt{|\mathbf{k}_i|^2} = \text{NA}. \tag{23}$$

Note the last equality holds because we are interested in the NA-matching angle illumination condition (Eq. 9) in this section (section 12.3). If we evaluate the CTF function H at $\mathbf{k}+\mathbf{k}_i$, we have $H(\mathbf{k}+\mathbf{k}_i) = \mathbb{1}(|\mathbf{k}+\mathbf{k}_i| \leq \text{NA})e^{j\phi(\mathbf{k})} = 0$ for all $\mathbf{k}$ subject to $\mathbf{k}_0\cdot\mathbf{e} = \mathbf{k}\cdot(-\mathbf{k}_i) < 0$. We can then prove Δ_i is one sided:

$$\hat{\Delta}_i(\mathbf{k}) = \hat{S}_i'(\mathbf{k})\big[1-\delta(\mathbf{k})\big]e^{-j\phi(\mathbf{k}_i)} = \hat{O}_i(\mathbf{k})H(\mathbf{k}+\mathbf{k}_i)\big[1-\delta(\mathbf{k})\big]e^{-j\phi(\mathbf{k}_i)} = 0, \quad \forall \mathbf{k} \text{ s.t. } \mathbf{k}\cdot(-\mathbf{k}_i) < 0. \tag{24}$$

Furthermore, for $\mathbf{k}\cdot\mathbf{e} = -\mathbf{k}\cdot\mathbf{k}_i = 0$, we have $\mathbf{k} \perp \mathbf{k}_i$. When $\mathbf{k} \neq \mathbf{0}$, the above two equations still hold. We only need to consider the special case, namely $\mathbf{k} = 0$. At $\mathbf{k} = 0$, we have

$$\hat{\Delta}_i(\mathbf{0}) = \hat{S}_i'(\mathbf{0})\big[1-\delta(\mathbf{0})\big]e^{-j\phi(\mathbf{k}_i)} = \hat{S}_i'(\mathbf{0})(1-1)e^{-j\phi(\mathbf{k}_i)} = 0. \tag{25}$$

That is, Δ_i is strictly one sided. Using lemma 12.2.1, we can conclude the Taylor series (the last term on the right-hand side of Eq. 17) is strictly one sided by induction.

Note that if one wants to directly use S_i instead, the "offset" field should be modified as $re^{2\pi j\mathbf{k}_i\cdot\mathbf{x}+j\phi(\mathbf{k}_i)}$. This is because the original model uses tilted illumination, so the unchanged light field is associated with an incident angle. With such modification, one can verify it gives an identical result.

Here, we have proved that the transformed signal $\log\left[S_i'(\mathbf{x})e^{-j\phi(\mathbf{k}_i)}\right]$ is one sided (It is easy to see that $\log\left[S_i'(\mathbf{x})\right]$ is also one sided, as $e^{-j\phi(\mathbf{k}_i)}$ is a constant). We proceed to show that such special structure allows one to reconstruct the imaginary part from the real part of the signal.

We now get more involved with a modified version of Eq. 10

$$\begin{aligned}\log\left[S_i'(\mathbf{x})e^{-j\phi(\mathbf{k}_i)}\right] &= \log\left[\left|S_i'(\mathbf{x})e^{-j\phi(\mathbf{k}_i)}\right|\right] + j\arg\left[S_i'(\mathbf{x})e^{-j\phi(\mathbf{k}_i)}\right] \\ &= \log\left[\left|S_i'(\mathbf{x})\right|\right] \qquad + j\Big(\arg\left[S_i'(\mathbf{x})\right] - \phi(\mathbf{k}_i)\Big) \\ &= \log(r) + \Re\big[T(\mathbf{x})\big] \quad + j\Im\big[T(\mathbf{x})\big],\end{aligned} \tag{26}$$

where the last equality follows from Eq. 17, $\Re(\cdot)$ denotes the real part of a complex number, and $\Im(\cdot)$ denotes the imaginary part. The Fourier transform exhibits even symmetric for a real signal and odd symmetric for an imaginary signal, so we have

$$\big[\mathcal{F}\big(\log|S_i'|\big)\big](\mathbf{k}) = \big[\mathcal{F}\big(\log|S_i'|\big)\big]^*(-\mathbf{k}), \tag{27}$$

and

$$\Big[\mathcal{F}\Big(j\big[\arg(S_i') - \phi(\mathbf{k}_i)\big]\Big)\Big](\mathbf{k}) = -\Big[\mathcal{F}\Big(j\big[\arg(S_i') - \phi(\mathbf{k}_i)\big]\Big)\Big]^*(-\mathbf{k}), \tag{28}$$

where $*$ in the superscript denotes complex conjugate. Because we have already proved that $\log\left[S_i'(\mathbf{x})e^{-j\phi(\mathbf{k}_i)}\right]$ is one sided, we have

$$\begin{aligned}&\Big[\mathcal{F}\Big(\log[S_i'e^{-j\phi(\mathbf{k}_i)}]\Big)\Big](\mathbf{k}) = \big[\mathcal{F}\big(\log|S_i'|\big)\big](\mathbf{k}) + \Big[\mathcal{F}\Big(j\big[\arg(S_i') - \phi(\mathbf{k}_i)\big]\Big)\Big](\mathbf{k}) = 0 \\ &\Rightarrow \Big[\mathcal{F}\Big(j\big[\arg(S_i') - \phi(\mathbf{k}_i)\big]\Big)\Big](\mathbf{k}) = -\big[\mathcal{F}\big(\log|S_i'|\big)\big](\mathbf{k}), \quad \forall\mathbf{k} \text{ s.t. } \mathbf{k}\cdot(-\mathbf{k}_i) < 0.\end{aligned} \tag{29}$$

For $\mathbf{k}\cdot(-\mathbf{k}_i) > 0$, we can use symmetry to conclude

$$\Big[\mathcal{F}\Big(j\big[\arg(S_i') - \phi(\mathbf{k}_i)\big]\Big)\Big](\mathbf{k}) = \big[\mathcal{F}\big(\log|S_i'|\big)\big](\mathbf{k}), \quad \forall\mathbf{k} \text{ s.t. } \mathbf{k}\cdot(-\mathbf{k}_i) > 0. \tag{30}$$

As $T(\mathbf{x})$ is strictly one sided, its Fourier transform is zero for all $\mathbf{k}$ such that $\mathbf{k}\perp\mathbf{k}_i$. Moreover, the Fourier transform of constant $\log(r)$ is a real (Dirac delta) function centered at zero. In other words, the Fourier transform of $\log(r)$ has no imaginary part. So, we have

$$\Big[\mathcal{F}\Big(j\big[\arg(S_i') - \phi(\mathbf{k}_i)\big]\Big)\Big](\mathbf{k}) = 0, \quad \forall\mathbf{k} \text{ s.t. } \mathbf{k}\cdot(-\mathbf{k}_i) = 0. \tag{31}$$

By collecting all pieces from the above three equations and noticing $I_i(\mathbf{x}) = \left|S_i'(\mathbf{x})\right|^2$, we have

$$\Big[\mathcal{F}\Big(\log[S_i'e^{-j\phi(\mathbf{k}_i)}]\Big)\Big](\mathbf{k}) = \begin{cases} 2\big[\mathcal{F}\big(\log|S_i'|\big)\big](\mathbf{k}) = \big[\mathcal{F}\big(\log I_i\big)\big](\mathbf{k}), & \mathbf{k}\cdot\mathbf{k}_i < 0, \\ \big[\mathcal{F}\big(\log|S_i'|\big)\big](\mathbf{k}) = \frac{1}{2}\big[\mathcal{F}\big(\log I_i\big)\big](\mathbf{k}), & \mathbf{k}\cdot\mathbf{k}_i = 0, \\ 0, & \mathbf{k}\cdot\mathbf{k}_i > 0. \end{cases} \tag{32}$$

That is, the complex field can be reconstructed using its real part $\log|S_i'|$. We can then restore the desired field (up to a constant phase offset) with inverse Fourier transform and apply exponential function to each point of the inverse Fourier transform

$$S_i'(\mathbf{x})e^{-j\phi(\mathbf{k}_i)} = \exp\left[\left(\mathcal{F}^{-1}\Big[\mathcal{F}\Big(\log[S_i'e^{-j\phi(\mathbf{k}_i)}]\Big)\Big]\right)(\mathbf{x})\right]. \tag{33}$$

Note that if there is no aberration in the system $\phi(\mathbf{k}) \equiv 0$, we have $S_i'(\mathbf{x}) = \hat{O}(\mathbf{k})\,\mathrm{Circ}_{\mathrm{NA}}(\mathbf{k}+\mathbf{k}_i)$ and the phase offset $\phi(\mathbf{k}_i) = 0$, which reduces to the result reported in previous literature [2, 9, 10].

12.3 Aberration extraction

In Section 12.2, we end up with the reconstruction of $S'_i(\mathbf{x})e^{-j\phi(\mathbf{k}_i)}$. Now we take a close look at its spectrum:

$$\hat{S}'_i(\mathbf{k})e^{-j\phi(\mathbf{k}_i)} = \hat{O}(\mathbf{k})H(\mathbf{k}+\mathbf{k}_i)e^{-j\phi(\mathbf{k}_i)} = \hat{A}(\mathbf{k})e^{j\hat{\alpha}(\mathbf{k})}\,\mathrm{Circ}_{\mathrm{NA}}(\mathbf{k}+\mathbf{k}_i)e^{j\phi(\mathbf{k}+\mathbf{k}_i)-j\phi(\mathbf{k}_i)}. \tag{34}$$

From this equation, we can see the sample's spectrum and the system's aberration function are superimposed in the reconstruction. For a practical optical system with aberration, we have not yet reconstructed a clean sample spectrum. Instead, we reconstructed an aberrated version of the desired spectrum.

Without correction, aberrations of an imaging system, including defocus due to sample's height unevenness, would largely degrade the reconstruction quality. Nonetheless, we will show that this problem can be tackled by working in the spatial frequency domain (that is, working with the spectrum). We prove that the aberration of the system is analytically solvable by considering the phases of the reconstructed spectrum.

To extract the aberration from this reconstructed spectrum, we will need a method to separate the contribution from the sample itself and the imaging system. We can achieve this by considering the phases of multiple reconstructed spectrums. We first define the overlap of two spectrums.

Definition 12.3.1. *For two reconstructed spectrums $\hat{S}'_i(\mathbf{k})$ and $\hat{S}'_l(\mathbf{k})$, we define a set $\mathcal{C}_{il}$ as*

$$\mathcal{C}_{il} \coloneqq \big\{\mathbf{k}\in\mathbb{R}^2 \big| H(\mathbf{k}+\mathbf{k}_i)H(\mathbf{k}+\mathbf{k}_l)\neq 0\big\}. \tag{35}$$

We say this two spectrums are overlapped if the set $\mathcal{C}_{il}$ is nonempty $\mathcal{C}_{il}\neq\emptyset$, and the overlap between $\hat{S}'_i(\mathbf{k})$ and $\hat{S}'_l(\mathbf{k})$ is $\mathcal{C}_{il}$.

Let us consider two spectrums $\hat{S}'_i(\mathbf{k})$ and $\hat{S}'_l(\mathbf{k})$ with (nonempty) overlap $\mathcal{C}_{il}\neq\emptyset$. We calculate their phase difference within the overlapped region $\mathcal{C}_{il}$. This gives

$$\begin{aligned}
&\arg\big[\hat{S}'_i(\mathbf{k})e^{-j\phi(\mathbf{k}_i)}\big] - \arg\big[\hat{S}'_l(\mathbf{k})e^{-j\phi(\mathbf{k}_l)}\big] \\
&\qquad = \big[\hat{\alpha}(\mathbf{k})+\phi(\mathbf{k}+\mathbf{k}_i)-\phi(\mathbf{k}_i)\big] - \big[\hat{\alpha}(\mathbf{k})+\phi(\mathbf{k}+\mathbf{k}_l)-\phi(\mathbf{k}_l)\big] \\
&\qquad = \phi(\mathbf{k}+\mathbf{k}_i)-\phi(\mathbf{k}_i)-\phi(\mathbf{k}+\mathbf{k}_l)+\phi(\mathbf{k}_l), \\
&\qquad = \big[\phi(\mathbf{k}+\mathbf{k}_i)-\phi(\mathbf{k}+\mathbf{k}_l)\big] - \big[\phi(\mathbf{k}_i)-\phi(\mathbf{k}_l)\big], \quad \text{if } \mathbf{k}\in\mathcal{C}_{il}.
\end{aligned} \tag{36}$$

We will refer the first term $\phi(\mathbf{k}+\mathbf{k}_i)-\phi(\mathbf{k}+\mathbf{k}_l)$ on the right-hand side as the aberration difference, and the last term $\phi(\mathbf{k}_i)-\phi(\mathbf{k}_l)$ as the offset. When we consider the phase difference of the two spectrums, the contribution from the sample spectrum cancels out and the difference depends solely on the system's aberration. We can easily see that the remaining phase difference is linear with respect to the aberration function. As such, we can construct a linear operator that maps the aberration into phase differences of two overlapping spectrums. To do so, we first rearrange the 2D spectrum into a vector.

Definition 12.3.2. *Assume $\mathbf{B}$ is a $m\times t$ matrix, we define a "flattening" operator $\mathrm{Flat}_{m,t}$ which concatenates every column of $\mathbf{B}$ and produces a vector of length mt. If we use $\boldsymbol{\mathcal{B}}$ to denote this flattened vector, we have*

$$\boldsymbol{\mathcal{B}} = \mathrm{Flat}_{m,t}(\mathbf{B}) \in \mathbb{R}^{mt\times 1} \quad \textit{and} \quad \boldsymbol{\mathcal{B}}[i_1+(i_2-1)m] = \mathbf{B}(i_1,i_2). \tag{37}$$

We also define an inverse operator $\mathrm{Flat}^{-1}_{m,t}$, which restores the matrix when applied to the flattened vector

$$\mathbf{B} = \mathrm{Flat}^{-1}_{m,t}(\boldsymbol{\mathcal{B}}). \tag{38}$$

Assume the sample spectrum we acquired is a $N\times N$ matrix, we write $\hat{\mathbf{S}}'_i\in\mathbb{C}^{N\times N}$ as the matrix form of the sampled spectrum, and $\boldsymbol{\phi}\in\mathbb{R}^{N\times N}$ as the matrix form of the aberration. The corresponding flattened version is then $\hat{\boldsymbol{\mathcal{S}}}'_i = \mathrm{Flat}_{N,N}(\hat{\mathbf{S}}'_i)\in\mathbb{C}^{N^2\times 1}$, and $\boldsymbol{\varphi} = \mathrm{Flat}_{N,N}(\boldsymbol{\phi})\in\mathbb{R}^{N^2\times 1}$, respectively.

We let $\mathbf{K}_u,\mathbf{K}_v\in\mathbb{R}^{N\times N}$ be the spatial frequency grid (with zero frequency at $[c_0,c_0] = \big(\lceil\frac{N}{2}\rceil,\lceil\frac{N}{2}\rceil\big)$, where $\lceil\frac{N}{2}\rceil$ is the smallest integer that is larger than $\frac{N}{2}$) for the sampled spectrum, where u and v denotes two orthogonal direction. Let $\boldsymbol{K}_u,\boldsymbol{K}_v\in\mathbb{R}^{N^2\times 1}$ be their flattened vectors $\boldsymbol{K}_u = \mathrm{Flat}_{N,N}(\mathbf{K}_u)$ and $\boldsymbol{K}_v = \mathrm{Flat}_{N,N}(\mathbf{K}_v)$. For simplicity, we let $\boldsymbol{K}(m), m\in\{1,2,\cdots,N^2\}$ denotes k vector in grid, which is defined as

$$\boldsymbol{K}(m) \coloneqq \big[\boldsymbol{K}_u(m),\boldsymbol{K}_v(m)\big]. \tag{39}$$

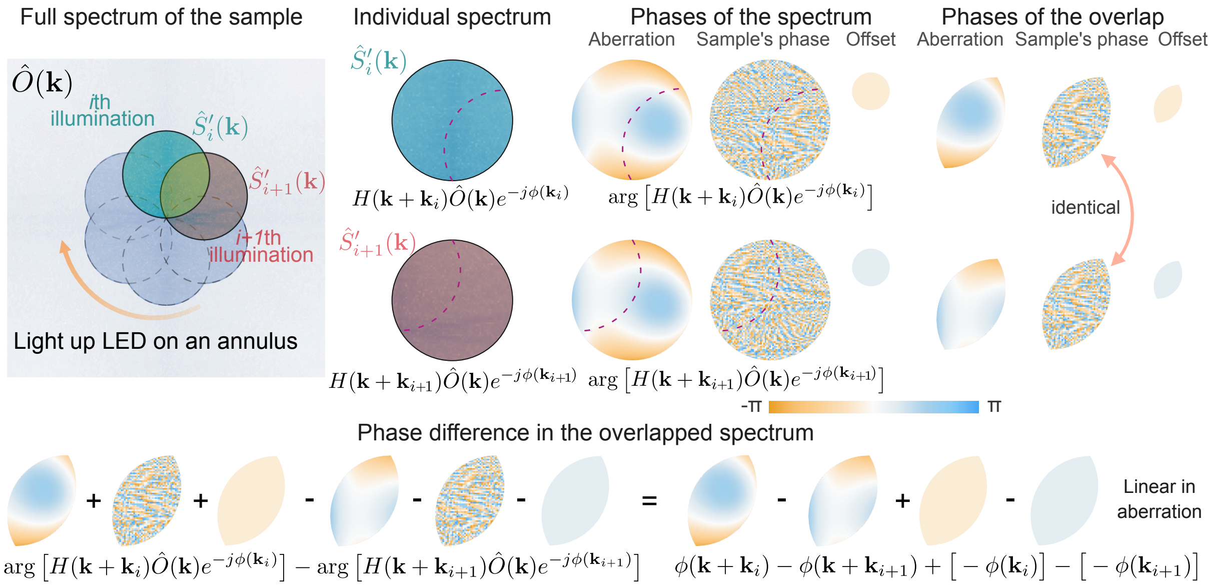

Figure S16: Phase difference of two spectrums with overlap. $\hat{O}(\mathbf{k})$ stands for the original sample's spectrum, $\hat{S}_i(\mathbf{k})$ is the acquired spectrum under ith illumination, and $H(\mathbf{k})$ is the coherent transfer function (CTF). For aberration extraction, we only use LED whose illumination k vector is matched with the maximal receiving angle of the imaging system $\mathbf{k}_i = \mathrm{NA}$. Here we select $l = i+1$ for illustration as the LEDs are lit up along an annulus shown in the figure. In the overlapped region, the phase of the object itself cancels out and phase difference of the shifted CTF solely contributes to the measured phase difference. This means the phase difference is linearly dependent on the aberration. Thus, we can design a operator that counts for the shift induced phase difference and use it to recover the imaging system's aberration.

Additionally, we define an index set $\boldsymbol{\mathcal{I}}_{il}$ by

$$\boldsymbol{\mathcal{I}}_{il} := \left\{ m = 1, 2, \cdots, N^2 \middle| \boldsymbol{K}(m) \in \mathcal{C}_{il} \right\}. \tag{40}$$

We let $|\boldsymbol{\mathcal{I}}_{il}|$ be the cardinal of set $\boldsymbol{\mathcal{I}}_{il}$ and also abuse the notation such that $\boldsymbol{\mathcal{I}}_{il}(m)$ indicate the mth smallest element in $\boldsymbol{\mathcal{I}}_{il}$. For the transverse illumination k-vector $\mathbf{k}_i$ $(i = 1, 2, \cdots, n)$, we denote its grid representation as $\boldsymbol{\kappa}_i$.

With this notation, we design a difference operator $\mathbf{D}_{il} \in \mathbb{R}^{|\boldsymbol{\mathcal{I}}_{il}| \times N^2}$. When acting on $\boldsymbol{\varphi}$, we want this operator to calculate the aberration difference $\phi(\mathbf{k} + \mathbf{k}_i) - \phi(\mathbf{k} + \mathbf{k}_l)$ associated with $\hat{\boldsymbol{\mathcal{S}}}'_i$ and $\hat{\boldsymbol{\mathcal{S}}}'_l$ (each row corresponds to one specific choice of $\mathbf{k}$). Based on this principle, we assign value 1 to the place that corresponds to phase $\phi(\mathbf{k} + \mathbf{k}_i)$ and value -1 to the place that corresponds to phase $\phi(\mathbf{k} + \mathbf{k}_l)$ for every row of $\mathbf{D}_{il}$. That is to say, the operator $\mathbf{D}_{il}$ is constructed as

$$\mathbf{D}_{il}(i_1, i_2) = \begin{cases} 1, & \text{if } \boldsymbol{K}(i_2) = \boldsymbol{K}\left[\boldsymbol{\mathcal{I}}_{il}(i_1)\right] + \boldsymbol{\kappa}_i \\ -1, & \text{if } \boldsymbol{K}(i_2) = \boldsymbol{K}\left[\boldsymbol{\mathcal{I}}_{il}(i_1)\right] + \boldsymbol{\kappa}_l \\ 0, & \text{otherwise.} \end{cases} \tag{41}$$

We can see this operator indeed calculates the aberration difference

$$\mathbf{D}_{il}\boldsymbol{\varphi} = \begin{bmatrix} \phi\Big(\boldsymbol{K}\left[\boldsymbol{\mathcal{I}}_{il}(1)\right] + \boldsymbol{\kappa}_i\Big) - \phi\Big(\boldsymbol{K}\left[\boldsymbol{\mathcal{I}}_{il}(1)\right] + \boldsymbol{\kappa}_l\Big) \\ \phi\Big(\boldsymbol{K}\left[\boldsymbol{\mathcal{I}}_{il}(2)\right] + \boldsymbol{\kappa}_i\Big) - \phi\Big(\boldsymbol{K}\left[\boldsymbol{\mathcal{I}}_{il}(2)\right] + \boldsymbol{\kappa}_l\Big) \\ \vdots \\ \phi\Big(\boldsymbol{K}\left[\boldsymbol{\mathcal{I}}_{il}(|\boldsymbol{\mathcal{I}}_{il}|)\right] + \boldsymbol{\kappa}_i\Big) - \phi\Big(\boldsymbol{K}\left[\boldsymbol{\mathcal{I}}_{il}(|\boldsymbol{\mathcal{I}}_{il}|)\right] + \boldsymbol{\kappa}_l\Big) \end{bmatrix}. \tag{42}$$

Let $\hat{\boldsymbol{\mathcal{S}}}_i^{'}[\boldsymbol{\mathcal{I}}_{il}]$ $(i = 1, 2, \cdots, n)$ be a $\mathbb{R}^{|\boldsymbol{\mathcal{I}}_{il}|\times 1}$ vector which is defined as

$$\hat{\boldsymbol{\mathcal{S}}}_i^{'}[\boldsymbol{\mathcal{I}}_{il}] \coloneqq \Big[\hat{\boldsymbol{\mathcal{S}}}_i^{'}[\boldsymbol{\mathcal{I}}_{il}(1)], \quad \hat{\boldsymbol{\mathcal{S}}}_i^{'}[\boldsymbol{\mathcal{I}}_{il}(2)], \quad \cdots, \quad \hat{\boldsymbol{\mathcal{S}}}_i^{'}[\boldsymbol{\mathcal{I}}_{il}(|\boldsymbol{\mathcal{I}}_{il}|)]\Big]^T, \tag{43}$$

where T in the superscript denotes ordinary transpose. We can then simplify the expression of $\mathbf{D}_{il}\boldsymbol{\varphi}$

$$\mathbf{D}_{il}\boldsymbol{\varphi} = \arg\Big(\hat{\boldsymbol{\mathcal{S}}}_i^{'}[\boldsymbol{\mathcal{I}}_{il}]\Big) - \arg\Big(\hat{\boldsymbol{\mathcal{S}}}_l^{'}[\boldsymbol{\mathcal{I}}_{il}]\Big) + \big[\phi(\mathbf{k}_i) - \phi(\mathbf{k}_l)\big]. \tag{44}$$

Note $\phi(\mathbf{k}_i) - \phi(\mathbf{k}_l)$ is a constant. To count for this constant, we define an offset operator $\mathbf{D}_{il}^0 \in \mathbb{R}^{|\boldsymbol{\mathcal{I}}_{il}|\times N^2}$, where we assign 1 to place that corresponds to $\mathbf{k}_i$ and -1 to $\mathbf{k}_l$. That is,

$$\mathbf{D}_{il}^0(i_1, i_2) = \begin{cases} 1, & \text{if } \boldsymbol{K}(i_2) = \boldsymbol{\kappa}_i \\ -1, & \text{if } \boldsymbol{K}(i_2) = \boldsymbol{\kappa}_l \\ 0, & \text{otherwise.} \end{cases} \tag{45}$$

When acting on $\boldsymbol{\varphi}$, this offset operator gives

$$\mathbf{D}_{il}^0\boldsymbol{\varphi} = \begin{bmatrix} \phi(\mathbf{k}_i) - \phi(\mathbf{k}_l) \\ \phi(\mathbf{k}_i) - \phi(\mathbf{k}_l) \\ \vdots \\ \phi(\mathbf{k}_i) - \phi(\mathbf{k}_l) \end{bmatrix} \tag{46}$$

Thus, we can use $\mathbf{D}_{il}$ and $\mathbf{D}_{il}^0$ to express the total phase difference between $\hat{\boldsymbol{\mathcal{S}}}_i^{'}$ and $\hat{\boldsymbol{\mathcal{S}}}_l^{'}$, which gives

$$(\mathbf{D}_{il} - \mathbf{D}_{il}^0)\boldsymbol{\varphi} = \arg\Big(\hat{\boldsymbol{\mathcal{S}}}_i^{'}[\boldsymbol{\mathcal{I}}_{il}]\Big) - \arg\Big(\hat{\boldsymbol{\mathcal{S}}}_l^{'}[\boldsymbol{\mathcal{I}}_{il}]\Big). \tag{47}$$

For different pairs of spectrums with overlap, we concatenate those equations, which yields

$$\mathbf{D}\boldsymbol{\varphi} = \boldsymbol{\beta}_\Delta,$$

where

$$\mathbf{D} \coloneqq \begin{bmatrix} \mathbf{D}_{i_1 l_1} - \mathbf{D}_{i_1 l_1}^0 \\ \mathbf{D}_{i_2 l_2} - \mathbf{D}_{i_2 l_2}^0 \\ \vdots \\ \mathbf{D}_{i_m l_m} - \mathbf{D}_{i_m l_m}^0 \end{bmatrix} \quad \text{and} \quad \boldsymbol{\beta}_\Delta \coloneqq \arg\begin{pmatrix} \hat{\boldsymbol{\mathcal{S}}}_{i_1}^{'}[\boldsymbol{\mathcal{I}}_{i_1 l_1}] \\ \hat{\boldsymbol{\mathcal{S}}}_{i_2}^{'}[\boldsymbol{\mathcal{I}}_{i_2 l_2}] \\ \vdots \\ \hat{\boldsymbol{\mathcal{S}}}_{i_m}^{'}[\boldsymbol{\mathcal{I}}_{i_m l_m}] \end{pmatrix} - \arg\begin{pmatrix} \hat{\boldsymbol{\mathcal{S}}}_{l_1}^{'}[\boldsymbol{\mathcal{I}}_{i_1 l_1}] \\ \hat{\boldsymbol{\mathcal{S}}}_{l_2}^{'}[\boldsymbol{\mathcal{I}}_{i_2 l_2}] \\ \vdots \\ \hat{\boldsymbol{\mathcal{S}}}_{l_m}^{'}[\boldsymbol{\mathcal{I}}_{i_m l_m}] \end{pmatrix} \tag{48}$$

Instead of directly solving for the aberration term, we use Zernike polynomial to represent the aberration of the system. Thus, we introduce the Zernike operator $\mathbf{Z} \in \mathbb{R}^{N^2\times z}$, where z is the number of Zernike coefficients to be reconstructed and each column of $\mathbf{Z}$ represents a particular Zernike mode. With Zernike decomposition, the aberration of the imaging system is given by

$$\boldsymbol{\varphi} = \mathbf{Z}\mathbf{c}, \tag{49}$$

where $\mathbf{c} \in \mathbb{R}^{z\times 1}$ is the corresponding Zernike coefficient. Using Zernike decomposition, we can rewrite Eq. 48 as

$$\boldsymbol{\beta}_\Delta = \mathbf{D}\boldsymbol{\varphi} = \mathbf{D}\mathbf{Z}\mathbf{c}. \tag{50}$$

We can solve the above linear equation (or the associated normal equation) to get the analytical solution of the Zernike coefficient $\mathbf{c}$. The 2D aberration $\boldsymbol{\phi}$ of the imaging system is then given by

$$\boldsymbol{\phi} = \text{Flat}_{N,N}^{-1}(\boldsymbol{\varphi}) = \text{Flat}_{N,N}^{-1}(\mathbf{Z}\mathbf{c}). \tag{51}$$

In reality, some spatial frequencies in the spectrum can be stronger than other frequencies. It is natural to emphasize on those places, as they have a higher signal-to-noise ratio (SNR). Thus, we use a weight matrix $\mathbf{W}$ to emphasize places with high SNR. This gives

$$(\mathbf{W}\mathbf{D}\mathbf{Z})\mathbf{c} = \mathbf{W}\boldsymbol{\beta}_\Delta. \tag{52}$$

In our experiment, we use logarithm of the modulus of the product $\hat{S}_i(\mathbf{k})\hat{S}_l(\mathbf{k})$ as the weight matrix. Another important thing to note is that the phase difference of two spectrums might exceed 2π. Thus, we first unwrap their phase differences [12] and then solve Eq. 52 to extract the aberration of our imaging system.

12.4 Reconstruction using darkfield measurements

Since aberrations of our imaging system are fully determined, we can remove the contribution of the aberration term from the reconstructed spectrums in section 12.2. Recall that in section 12.2, we have reconstructed the (modified) sampled spectrums (Eqs. 32 and 33) under NA-matching angle illumination. This means the following is known for all $|\mathbf{k}_i| = \mathrm{NA}$

$$\hat{S}_i'(\mathbf{k})e^{-j\phi(\mathbf{k}_i)} = \hat{O}(\mathbf{k})H(\mathbf{k}+\mathbf{k}_i)e^{-j\phi(\mathbf{k}_i)} = \hat{O}(\mathbf{k})\,\mathrm{Circ}_{\mathrm{NA}}(\mathbf{k}+\mathbf{k}_i)e^{j\phi(\mathbf{k}+\mathbf{k}_i)-j\phi(\mathbf{k}_i)}. \tag{53}$$

As the last phase factor $e^{j\phi(\mathbf{k}+\mathbf{k}_i)-j\phi(\mathbf{k}_i)}$ entirely depends on the aberration, we can now correct it and extract a clean sample spectrum, which yields

$$\hat{O}(\mathbf{k})\,\mathrm{Circ}_{\mathrm{NA}}(\mathbf{k}+\mathbf{k}_i). \tag{54}$$

It basically means that we recovered a piece of sample spectrum region covered by the CTF support for each measurement. Thus, these reconstructed regions can be stitched for a larger coverage in the spatial frequency domain. That is, we can gradually expand the coverage of the reconstructed $\hat{O}(\mathbf{k})$ in the spatial frequency domain. Let us define the sampled region $\mathcal{M}_m$ that denotes the spectrum covered by the first mth measurements

$$\mathcal{M}_m \coloneqq \big\{\mathbf{k}\in\mathbb{R}^2 \big| \exists i \in \{1,2,\cdots,m\}, \mathrm{Circ}_{\mathrm{NA}}(\mathbf{k}+\mathbf{k}_i) \neq 0\big\}. \tag{55}$$

We also define the mask M_m which denotes the effective sampling mask for the first mth measurements

$$M_m(\mathbf{k}) \coloneqq \mathbb{1}(\mathbf{k}\in\mathcal{M}_m) = \begin{cases} 1, & \text{if } \mathbf{k}\in\mathcal{M}_m, \\ 0, & \text{otherwise.}\end{cases} \tag{56}$$

Assume we can reconstruct the complex spectrum for every measurement (we will later show this is feasible), the reconstructed complex sample spectrum $\hat{\mathsf{R}}_m(\mathbf{k})$ using the first mth measurements can be expressed by

$$\hat{\mathsf{R}}_m(\mathbf{k}) \coloneqq \hat{O}(\mathbf{k})M_m(\mathbf{k}). \tag{57}$$

It is worth noting that, after aberration correction, we have obtained the reconstructed spectrum, $\hat{\mathsf{R}}_{n_0}(\mathbf{k}) = \hat{O}(\mathbf{k})M_{n_0}(\mathbf{k})$, using NA-matching measurements, $i = 1,2,\cdots,n_0$. For $i > n_0$, the corresponding illumination angle exceeds the maximal acceptance angle of the imaging system and we measure the darkfield (see Eq. 9 for the definition of n_0).

We note that the logarithm transformation technique used in section 12.2 fails as the darkfield may probably contain null point and the Taylor expansion might not converge. As a consequence, we need to develop another algorithm to reconstruct the sampled complex spectrum.

In this section, we show that if the sampled spectrum at ith illumination $\hat{S}_i'$ $(i > n_0)$ consists of previously reconstructed (*a priori*) spectrum and other unknown part, the unknown part can be reconstructed using the known spectrum.

Let us decompose the sampled spectrum $\hat{S}_i'$ into the unknown and known part, assuming all previous measurements are reconstructed, which means $\hat{\mathsf{R}}_{i-1}(\mathbf{k}) = \hat{O}(\mathbf{k})M_{i-1}(\mathbf{k})$ is known. The known spectrum $\hat{\mathcal{P}}_i(\mathbf{k})$ at this substep is given by

$$\hat{\mathcal{P}}_i(\mathbf{k}) = \hat{\mathsf{R}}_{i-1}(\mathbf{k})H(\mathbf{k}+\mathbf{k}_i) = \hat{O}(\mathbf{k})H(\mathbf{k}+\mathbf{k}_i)M_{i-1}(\mathbf{k}). \tag{58}$$

Then, the unknown part $\hat{U}_i(\mathbf{k})$ is

$$\begin{aligned}\hat{U}_i(\mathbf{k}) &= \hat{S}_i'(\mathbf{k}) - \hat{\mathcal{P}}_i(\mathbf{k}) \\ &= \hat{O}(\mathbf{k})H(\mathbf{k}+\mathbf{k}_i) - \hat{O}(\mathbf{k})H(\mathbf{k}+\mathbf{k}_i)M_{i-1}(\mathbf{k}) \\ &= \hat{O}(\mathbf{k})H(\mathbf{k}+\mathbf{k}_i)\big[1 - M_{i-1}(\mathbf{k})\big].\end{aligned} \tag{59}$$

Let $\mathrm{Supp}(f)$ be the support of function $f:\mathbb{R}^2\to\mathbb{C}$, which is a set given by

$$\mathrm{Supp}(f) \coloneqq \{\mathbf{x}\in\mathbb{R}^2 \big| f(\mathbf{x}) \neq 0\}. \tag{60}$$

By construction of $\hat{\mathcal{P}}_i(\mathbf{k})$ and $\hat{U}_i(\mathbf{k})$, it is easy to see that $\hat{\mathcal{P}}_i(\mathbf{k}) \neq 0$ suggests $\hat{U}_i(\mathbf{k}) = 0$, and vice versa. Thus, $\hat{\mathcal{P}}_i(\mathbf{k})$ and $\hat{U}_i(\mathbf{k})$ have disjoint support. Based on Eqs. 5 and 6, the measured intensity of one darkfield measurement can be expressed as

$$\begin{aligned} I_i(\mathbf{x}) &= \Big|\big[\mathcal{F}^{-1}(\hat{S}_i')\big](\mathbf{x})\Big|^2 = \Big|\big[\mathcal{F}^{-1}(\hat{U}_i + \hat{\mathcal{P}}_i)\big](\mathbf{x})\Big|^2 = \Big|U_i(\mathbf{x}) + \mathcal{P}_i(\mathbf{x})\Big|^2 \\ &= U_i(\mathbf{x})U_i^*(\mathbf{x}) + U_i(\mathbf{x})\mathcal{P}_i^*(\mathbf{x}) + \mathcal{P}_i(\mathbf{x})U_i^*(\mathbf{x}) + \mathcal{P}_i(\mathbf{x})\mathcal{P}_i^*(\mathbf{x}), \end{aligned} \tag{61}$$

where $\mathcal{P}(\mathbf{x}) \coloneqq \big[\mathcal{F}^{-1}(\hat{\mathcal{P}}_i\big](\mathbf{x})$ and $U(\mathbf{x}) \coloneqq \big[\mathcal{F}^{-1}(\hat{U}_i\big](\mathbf{x})$ are the known and unknown fields, respectively. Using the property of Fourier transform, we can write the Fourier transform of I_i as

$$\big[\mathcal{F}(I_i)\big](\mathbf{k}) = \big[\hat{U}_i \star \hat{U}_i\big](\mathbf{k}) + \big[\hat{U}_i \star \hat{\mathcal{P}}_i\big](\mathbf{k}) + \big[\hat{\mathcal{P}}_i \star \hat{U}_i\big](\mathbf{k}) + \big[\hat{\mathcal{P}}_i \star \hat{\mathcal{P}}_i\big](\mathbf{k}), \tag{62}$$

where $\star$ denotes correlation. As the known spectrum is *a priori*, we can subtract its auto-correlation from the Fourier transform of the intensity measurement. This yields

$$\big[\mathcal{F}(I_i)\big](\mathbf{k}) - \big[\hat{\mathcal{P}}_i \star \hat{\mathcal{P}}_i\big](\mathbf{k}) = \big[\hat{U}_i \star \hat{U}_i\big](\mathbf{k}) + \big[\hat{U}_i \star \hat{\mathcal{P}}_i\big](\mathbf{k}) + \big[\hat{\mathcal{P}}_i \star \hat{U}_i\big](\mathbf{k}). \tag{63}$$

We can see that the two cross-terms are linear with respect to $\hat{U}_i$ as correlation is a linear operator. If we consider one cross term, it naturally leads to a linear equation with respect to $\hat{U}_i$, which can be solved analytically. However, those three remaining terms cannot be easily separated due to the existence of our desired unknown part.

In the remaining part of this section, we see that the above three terms have different supports, as depicted in Fig. S17. Therefore, the non-overlapping part can be isolated, which allows us to construct a linear equation with respect to $\hat{U}_i$.

In experiments, the same camera is used to acquire both darkfield and NA-matching measurements. Each intensity measurement is a matrix of fixed size. Similar to the FPM, the matrix representation of the original sample's spectrum R_m is nevertheless a much larger matrix [3, 4]. The physical picture is that the tilt illumination translates the sample spectrum in the spatial frequency domain, as the initial forward model Eq. 1 suggests, so that different spatial frequencies of sample spectrum is moved into this smaller measurement related grid and then gets sampled. This is equivalent to cropping out a specific region of the sample spectrum and moving the cropped part to the center when applying the pupil translation model in Eq. 6. In our following discussion, we assume the matrix representations of both the unknown and the known spectrum are centered, thus having the same dimension as the measurement.

We focus on $\big[\hat{\mathcal{P}}_i \star \hat{U}_i\big](\mathbf{k})$ and let $\mathcal{Q}_i$ be the non-intersecting set, which is defined as

$$\mathcal{Q}_i \coloneqq \Big\{\mathbf{k} \in \mathbb{R}^2 \Big| \mathbf{k} \in \operatorname{Supp}(\hat{\mathcal{P}}_i \star \hat{U}_i) \setminus \big[\operatorname{Supp}(\hat{U}_i \star \hat{\mathcal{P}}_i) \cup \operatorname{Supp}(\hat{U}_i \star \hat{U}_i)\big]\Big\}. \tag{64}$$

By construction, we have

$$\big[\mathcal{F}(I_i)\big](\mathbf{k}) - \big[\hat{\mathcal{P}}_i \star \hat{\mathcal{P}}_i\big](\mathbf{k}) = \big[\hat{\mathcal{P}}_i \star \hat{U}_i\big](\mathbf{k}), \quad \forall \mathbf{k} \in \mathcal{Q}_i. \tag{65}$$

We define this masked subtraction as L_i, that is

$$L_i(\mathbf{k}) \coloneqq \Big(\big[\mathcal{F}(I_i)\big](\mathbf{k}) - \big[\hat{\mathcal{P}}_i \star \hat{\mathcal{P}}_i\big](\mathbf{k})\Big)\mathbb{1}(\mathbf{k} \in \mathcal{Q}_i) = \begin{cases} \big[\hat{\mathcal{P}}_i \star \hat{U}_i\big](\mathbf{k}), & \text{if } \mathbf{k} \in \mathcal{Q}_i, \\ 0, & \text{otherwise.} \end{cases} \tag{66}$$

Let $\hat{\mathbf{U}}_i \in \mathbb{C}^{N \times N}$ be the matrix version of the (centered) unknown part and $\hat{\boldsymbol{\mathcal{U}}}_i \in \mathbb{C}^{N^2 \times 1}$ be its flattened vector $\hat{\boldsymbol{\mathcal{U}}}_i = \operatorname{Flat}_{N,N}(\hat{\mathbf{U}}_i)$, $\mathbf{L}_i \in \mathbb{C}^{N \times N}$ be the (centered) matrix version of L_i and $\boldsymbol{\mathcal{L}}_i \in \mathbb{C}^{N^2 \times 1}$ be its flattened vector $\boldsymbol{\mathcal{L}}_i = \operatorname{Flat}_{N,N}(\mathbf{L}_i)$, and $\hat{\boldsymbol{\mathcal{P}}}_i$ be the (centered) matrix version of the known part. In general, the correlation of two matrices of size $N \times N$ would be a matrix of size $(2N-1) \times (2N-1)$. Based on the Nyquist theorem, the nonzero part of $\hat{\mathbf{U}}_i + \hat{\boldsymbol{\mathcal{P}}}_i$ is contained in a $\frac{N}{2} \times \frac{N}{2}$ box. As a consequence, we can calculate the correlation of $\hat{\mathbf{U}}_i$ and $\hat{\boldsymbol{\mathcal{P}}}_i$ using a $N \times N$ grid.

Let us construct a (sparse) correlation operator $\mathbf{C}_i$ that takes all nonzero elements in the unknown spectrum $\hat{\boldsymbol{\mathcal{U}}}_i$ and gives $\boldsymbol{\mathcal{L}}_i$. We first focus on the t_1th row and t_2th column of $\mathbf{L}_i$, which corresponds to mth element of vector $\boldsymbol{\mathcal{L}}_i$, where $m = t_1 + N(t_2 - 1)$ and $t_1, t_2 \in \{1, 2, \cdots, N\}$. Let $t_1' = t_1 - c_0$ and

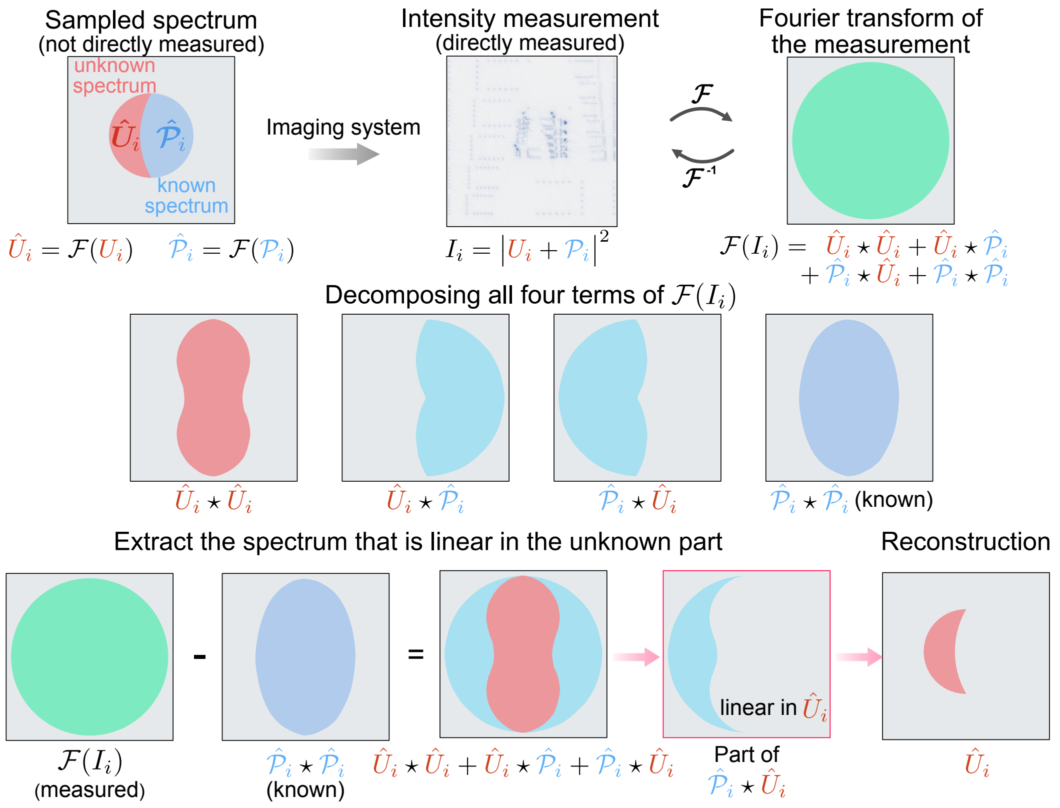

Figure S17: Complex field reconstruction using darkfield measurements. In this visualization, the sampled spectrum is centered, which is basically a cropped version of the spectrum translation model (Eq. 6). The sampled spectrum is decomposed into two disjoint region, namely the unknown and known spectrum. As the spectrum of the known part is given, the intensity of the corresponding field can be calculated. When subtracting the Fourier transform of the measured intensity and the Fourier transform of this calculated intensity, three term are left: the auto-correlation of the unknown part, and two cross-correlations of the known and unknown part. For one of the cross-correlation terms that does not overlap with the other two, we obtain a linear equation with respect to the unknown part. Thus, we can solve this linear equation for the closed-form solution of the unknown spectrum. That is, the complex spectrum can be reconstructed by incorporating the known information.

$t'_2 = t_2 - c_0$. Then, we have (recall that in Eq. 39 we define $\boldsymbol{K}$ as the grid version of the k vector and also $\mathbf{K}_u(c_0, c_0) = \mathbf{K}_v(c_0, c_0) = 0$)

$$\boldsymbol{\mathcal{L}}_i(m) = \begin{cases} \left[\hat{\boldsymbol{\mathcal{P}}}_i \star \hat{\mathbf{U}}_i\right]\left[\boldsymbol{K}(m)\right] & \text{if } \boldsymbol{K}(m) \in \mathcal{Q}_i \\ 0, & \text{otherwise} \end{cases}$$
$$= \begin{cases} \displaystyle\sum_{i_1=\max\{1,1+t'_1\}}^{\min\{N,N+t'_1\}} \sum_{i_2=\max\{1,1+t'_2\}}^{\min\{N,N+t'_2\}} \hat{\boldsymbol{\mathcal{P}}}^*_i(i_1 - t'_1, i_2 - t'_2)\hat{\mathbf{U}}_i(i_1, i_2), & \text{if } \boldsymbol{K}(m) \in \mathcal{Q}_i, \\ 0, & \text{otherwise.} \end{cases} \quad (67)$$

For $\boldsymbol{K}(m) \in \mathcal{Q}_i$, we can construct a matrix $\mathbf{G}_i^m \in \mathbb{C}^{N\times N}$ that is defined as

$$\mathbf{G}_i^m(i_1, i_2) := \begin{cases} \hat{\boldsymbol{\mathcal{P}}}^*_i(i_1 - t'_1, i_2 - t'_2), & \text{if } \max\{1, 1 + t'_\zeta\} \le i_\zeta \le \min\{N, N + t'_\zeta\}, \zeta \in \{1, 2\} \\ 0, & \text{otherwise.} \end{cases} \quad (68)$$

With this definition, we have

$$\boldsymbol{\mathcal{L}}_i(m) = \begin{cases} \left[\mathrm{Flat}_{N,N}(\mathbf{G}_i^m)\right]^T \hat{\boldsymbol{\mathcal{U}}}_i, & \text{if } \boldsymbol{K}(m) \in \mathcal{Q}_i \\ 0, & \text{otherwise.} \end{cases} \quad (69)$$

We define an index set $\mathfrak{L}_i$ that denotes this special region that is linear in $\hat{\boldsymbol{\mathcal{U}}}_i$

$$\mathfrak{L}_i \coloneqq \{m = 1, 2, \cdots, N^2 \big| \boldsymbol{K}(m) \in \mathcal{Q}_i\}. \tag{70}$$

As we have done before, we abuse the notation so that $\mathfrak{L}_i(m)$ indicates the mth smallest element in $\mathfrak{L}_i$ and define $\boldsymbol{\mathcal{L}}_i(\mathfrak{L}_i)$ as

$$\boldsymbol{\mathcal{L}}_i(\mathfrak{L}_i) \coloneqq \Big[\boldsymbol{\mathcal{L}}_i\big[\mathfrak{L}_i(1)\big], \quad \boldsymbol{\mathcal{L}}_i\big[\mathfrak{L}_i(2)\big], \quad \cdots, \quad \boldsymbol{\mathcal{L}}_i\big[\mathfrak{L}_i(|\mathfrak{L}_i|)\big]\Big]^T, \tag{71}$$

where $|\mathfrak{L}_i|$ is the cardinal of $\mathfrak{L}_i$. We can then construct a correlation operator. Let $\mathbf{C}_i^F \in \mathbb{C}^{|\mathfrak{L}_i| \times N^2}$ be a $|\mathfrak{L}_i| \times N^2$ matrix and let its mth row $\mathbf{C}_i^F(m, \cdot)$ be

$$\mathbf{C}_i^F(m, \cdot) = \big[\text{Flat}_{N,N}(\mathbf{G}_i^{\mathfrak{L}_i(m)})\big]^T, \tag{72}$$

we then have

$$\mathbf{C}_i^F \hat{\boldsymbol{\mathcal{U}}}_i = \boldsymbol{\mathcal{L}}_i(\mathfrak{L}_i). \tag{73}$$

Graphically, the construction of the correlation operator $\mathbf{C}_i^F$ is shown in Fig. S18.

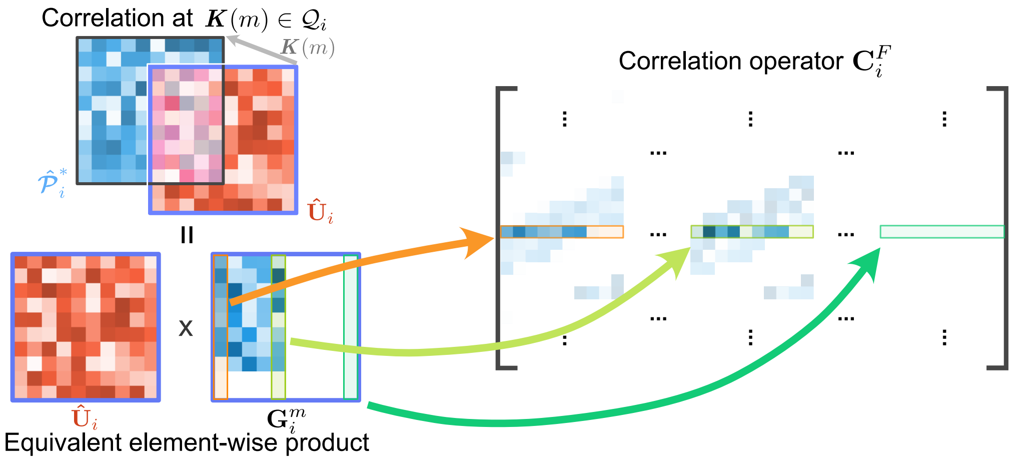

Figure S18: Construction of the correlation operator $\mathbf{C}_i^F$. For vector $\boldsymbol{K}(m)$ that admits a measurement that is linear in the unknown spectrum, we construct an associated matrix $\mathbf{G}_i^m$. This matrix is constructed such that the correlation of the unknown and known part at $\boldsymbol{K}(m)$ equals $\sum_{i_1,i_2=1}^N \hat{\mathbf{U}}_i(i_1, i_2)\mathbf{G}_i^m(i_1, i_2)$, the summation of the element-wise multiplication of this matrix and the unknown spectrum. The flattened vector of $\mathbf{G}_i^m$ serves as one row of the correlation matrix $\mathbf{C}_i^F$, as illustrated in the figure.

By construction of the unknown spectrum $\hat{U}_i$, we know that the only locations that it can be nonzero are where the following holds

$$H(\mathbf{k} + \mathbf{k}_i)\big[1 - M_{i-1}(\mathbf{k})\big] \neq 0. \tag{74}$$

Using the above equation, we can easily find the corresponding nonzero elements in the flattened vector $\hat{\boldsymbol{\mathcal{U}}}_i$. We define an index set $\mathbf{N}_i$ consists of the indices of these nonzero elements

$$\mathbf{N}_i \coloneqq \Big\{m = 1, 2, \cdots, N^2 \Big| H\big[\boldsymbol{K}(m) + \boldsymbol{\kappa}_i\big]\Big(1 - M_{i-1}\big[\boldsymbol{K}(m)\big]\Big) \neq 0\Big\}, \tag{75}$$

and let $\mathbf{N}_i(m)$ be the mth smallest element in $\mathbf{N}_i$, and $|\mathbf{N}_i|$ be the cardinal of $\mathbf{N}_i$. To construct the correlation operator $\mathbf{C}_i$ that encodes the sparsity of the unknown spectrum, we simply keep all columns of $\mathbf{C}_i^F$ whose indices belong to set $\mathbf{N}_i$ and throw away all other columns. This gives the definition of $\mathbf{C}_i \in \mathbb{C}^{|\mathfrak{L}_i| \times |\mathbf{N}_i|}$

$$\mathbf{C}_i(i_1, m) \coloneqq \mathbf{C}_i^F\big[i_1, \mathbf{N}_i(m)\big], \quad i_1 = 1, 2, \cdots, |\mathfrak{L}_i| \text{ and } m = 1, 2, \cdots, |\mathbf{N}_i|. \tag{76}$$

Then, we have the following linear equation with respect to the nonzero elements of the unknown spectrum

$$\mathbf{C}_i \hat{\boldsymbol{\mathcal{U}}}_i(\mathbf{N}_i) = \boldsymbol{\mathcal{L}}_i(\mathfrak{L}_i), \tag{77}$$

where $\hat{\boldsymbol{\mathcal{U}}}_i(\mathbf{N}_i) \coloneqq \Big[\hat{\boldsymbol{\mathcal{U}}}_i\big[\mathbf{N}_i(1)\big], \quad \hat{\boldsymbol{\mathcal{U}}}_i\big[\mathbf{N}_i(2)\big], \quad \cdots, \quad \hat{\boldsymbol{\mathcal{U}}}_i\big[\mathbf{N}_i(|\mathbf{N}_i|)\big]\Big]^T$.

To solve this equation, we require the rank of matrix $\mathbf{C}_i$ to be at least $|\mathbf{N}_i|$. We can show this can be satisfied if the known spectrum covers the semicircle of the circular CTF. As shown in Fig. S19, the autocorrelation of a semicircle is around 4 times larger than itself. If we assume the CTF is of radius r_0, and the known specturm is a semicircle, the area of the unknown spectrum is then $\frac{1}{2}\pi r_0^2$. For a circle (area is πr_0^2), its autocorrelation is strictly 4 times larger in size (area is $4\pi r_0^2$). Thus, the linear region has an area of

$$\mathrm{Area}(\mathcal{Q}_i) = \frac{1}{2}\big[4\pi r_0^2 - (4r_0^2 + \pi r_0^2)\big] = \frac{3\pi r_0^2 - 4r_0^2}{2}, \tag{78}$$

which is approximately 1.7 times larger than the area of the unknown part. That is, if the known spectrum occupies 50% of the spectrum, the rank of matrix $\mathbf{C}_i$ can be well above $|\mathbf{N}_i|$. Numerically, we find a safe choice is to let the unknown spectrum occupy over 42% of the measured spectrum, assume the CTF is circular. We note that resultant shape of the correlation of two complex objects is nontrivial as thus it is beneficial if to determine the shape of the aperture before the actual measurement and use it as a prior knowledge in the reconstruction. We focus on the circular CTF in this section as it is the most common case.

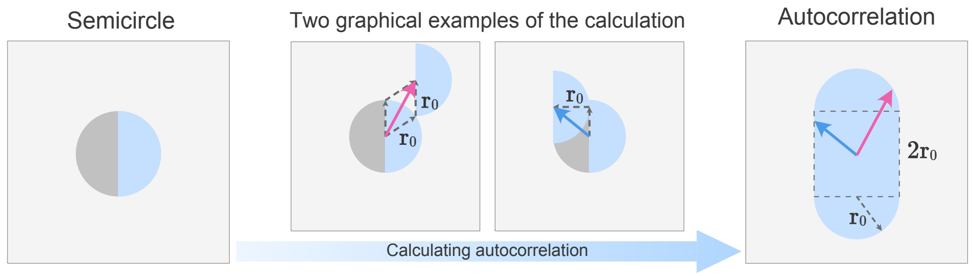

Figure S19: Autocorrelation of a semicircle with radius r_0. The shape of the autocorreltion result has an area of $4r_0^2 + \pi r_0^2$ which is around 4.5 times larger than the area enclosed by the semicircle.

By solving Eq. 77, we obtain the closed-form solution of the unknown spectrum. That is, we reconstruct the following spectrum

$$\hat{U}_i(\mathbf{k}) = \hat{O}(\mathbf{k})H(\mathbf{k}+\mathbf{k}_i)\big[1 - M_{i-1}(\mathbf{k})\big]. \tag{79}$$

As the aberration of the system is determined, we can correct for the aberration, which gives the aberration corrected spectrum $\hat{O}(\mathbf{k})\,\mathrm{Circ}_{\mathrm{NA}}(\mathbf{k}+\mathbf{k}_i)\big[1-M_{i-1}(\mathbf{k})\big]$. Because the intensity of the field is directly measured in the darkfield measurement and the phase is unknown, we use the square root of this measured intensity as the modulus of the complex field for maintaining the (point-wise) energy and robustness.

Until now, we show that the complex spectrum sampled with ith illumination can be reconstructed using *a priori* knowledge of the known spectrum. We can expand the entire reconstructed spectrum by integrating this newly reconstructed spectrum. The overall reconstructed spectrum using ith measurement is given by $\hat{O}(\mathbf{k})M_{i-1}(\mathbf{k}) + \hat{O}(\mathbf{k})\,\mathrm{Circ}_{\mathrm{NA}}(\mathbf{k}+\mathbf{k}_i)\big[1 - M_{i-1}(\mathbf{k})\big]$, which is exactly $\hat{O}(\mathbf{k})M_i(\mathbf{k})$ by definition of $M_i(\mathbf{k})$. That is, we obtain an extended complex spectrum reconstuction $\hat{\mathsf{R}}_i$ after darkfield reconstruction at the ith sub-step. This reconstructed field serves as our *a priori* knowledge in the reconstruction of the $(i+1)$th sub-step.

We note that for a dense LED array, the area of the unknown spectrum can be quite small because their effective CTFs cover similar area for two closely spaced LEDs (Eq. 6). In such case, we can fill in the new spectrum when it is necessary to solve the linear equation formed in Eq. 77 (e.g. when the system becomes undetermined if we do not fill in the new spectrum). By doing this, the spectrum reconstructions prior to the stitching process are independent and they have overlaps in the spatial frequency domain. Therefore, we can average over the overlap for improved robustness of the reconstruction algorithm.

Once the darkfield reconstruction is done for all measurements, we have reconstructed all sampled spectrums and the extended complex spectrum $\hat{\mathsf{R}}_n$, which is our high-resolution, aberration-free complex field reconstruction.

References

1. Eckert, R., Phillips, Z. F. & Waller, L. Efficient illumination angle self-calibration in Fourier ptychography. *Applied Optics* **57.** Publisher: Optica Publishing Group, 5434–5442. ISSN: 2155-3165. https://opg.optica.org/ao/abstract.cfm?uri=ao-57-19-5434 (2018).
2. Shen, C., Liang, M., Pan, A. & Yang, C. Non-iterative complex wave-field reconstruction based on Kramers–Kronig relations. *Photonics Research* **9.** Publisher: Optica Publishing Group, 1003–1012. ISSN: 2327-9125. https://opg.optica.org/prj/abstract.cfm?uri=prj-9-6-1003 (2021).
3. Zheng, G., Horstmeyer, R. & Yang, C. Wide-field, high-resolution Fourier ptychographic microscopy. *Nature Photonics* **7,** 739–745. ISSN: 1749-4893. https://www.nature.com/articles/nphoton.2013.187 (2013).
4. Zheng, G., Shen, C., Jiang, S., Song, P. & Yang, C. Concept, implementations and applications of Fourier ptychography. *Nature Reviews Physics* **3.** Number: 3 Publisher: Nature Publishing Group, 207–223. ISSN: 2522-5820. https://www.nature.com/articles/s42254-021-00280-y (2021).
5. Ou, X., Zheng, G. & Yang, C. Embedded pupil function recovery for Fourier ptychographic microscopy. *Optics Express* **22.** Publisher: Optica Publishing Group, 4960–4972. ISSN: 1094-4087. https://opg.optica.org/oe/abstract.cfm?uri=oe-22-5-4960 (2014).
6. Tian, L., Li, X., Ramchandran, K. & Waller, L. Multiplexed coded illumination for Fourier Ptychography with an LED array microscope. *Biomedical Optics Express* **5.** Publisher: Optica Publishing Group, 2376–2389. ISSN: 2156-7085. https://opg.optica.org/boe/abstract.cfm?uri=boe-5-7-2376 (2014).
7. Tian, L. *et al.* Computational illumination for high-speed in vitro Fourier ptychographic microscopy. *Optica* **2,** 904–911. ISSN: 2334-2536. https://www.osapublishing.org/optica/abstract.cfm?uri=optica-2-10-904 (2015).
8. Yeh, L.-H. *et al.* Experimental robustness of Fourier ptychography phase retrieval algorithms. *Optics Express* **23.** Publisher: Optica Publishing Group, 33214–33240. ISSN: 1094-4087. https://opg.optica.org/oe/abstract.cfm?uri=oe-23-26-33214 (2015).
9. Baek, Y. & Park, Y. Intensity-based holographic imaging via space-domain Kramers–Kronig relations. *Nature Photonics* **15.** Number: 5 Publisher: Nature Publishing Group, 354–360. ISSN: 1749-4893. https://www.nature.com/articles/s41566-021-00760-8 (2021).
10. Baek, Y., Lee, K., Shin, S. & Park, Y. Kramers–Kronig holographic imaging for high-space-bandwidth product. *Optica* **6.** Publisher: Optica Publishing Group, 45–51. ISSN: 2334-2536. https://opg.optica.org/optica/abstract.cfm?uri=optica-6-1-45 (2019).
11. Havlicek, J., Havlicek, J. & Bovik, A. *The analytic image* in *Proceedings of International Conference on Image Processing* Proceedings of International Conference on Image Processing. **2** (1997), 446–449 vol.2.
12. Ghiglia, D. C. & Romero, L. A. Robust two-dimensional weighted and unweighted phase unwrapping that uses fast transforms and iterative methods. *JOSA A* **11.** Publisher: Optica Publishing Group, 107–117. ISSN: 1520-8532. https://opg.optica.org/josaa/abstract.cfm?uri=josaa-11-1-107 (1994).
